# Widespread subclinical cellular changes revealed across a neural-epithelial-vascular complex in choroideremia using adaptive optics

**DOI:** 10.1038/s42003-022-03842-7

**Published:** 2022-09-13

**Authors:** Nancy Aguilera, Tao Liu, Andrew J. Bower, Joanne Li, Sarah Abouassali, Rongwen Lu, John Giannini, Maximilian Pfau, Chelsea Bender, Margery G. Smelkinson, Amelia Naik, Bin Guan, Owen Schwartz, Andrei Volkov, Alfredo Dubra, Zhuolin Liu, Daniel X. Hammer, Dragan Maric, Robert Fariss, Robert B. Hufnagel, Brett G. Jeffrey, Brian P. Brooks, Wadih M. Zein, Laryssa A. Huryn, Johnny Tam

**Affiliations:** 1 National Eye Institute, National Institutes of Health, Bethesda, MD USA; 2 National Institute of Allergy and Infectious Disease, Research Technologies Branch, National Institutes of Health, Bethesda, MD USA; 3 Department of Ophthalmology, Stanford University, Palo Alto, CA USA; 4 Division of Biomedical Physics, Office of Science and Engineering Laboratories, Center for Devices and Radiological Health, Food and Drug Administration, Silver Spring, MD USA; 5 National Institute of Neurological Disorders and Stroke, National Institutes of Health, Bethesda, MD USA

**Keywords:** Retinal diseases, Hereditary eye disease, Optical imaging, Fluorescence imaging

## Abstract

Choroideremia is an X-linked, blinding retinal degeneration with progressive loss of photoreceptors, retinal pigment epithelial (RPE) cells, and choriocapillaris. To study the extent to which these layers are disrupted in affected males and female carriers, we performed multimodal adaptive optics imaging to better visualize the in vivo pathogenesis of choroideremia in the living human eye. We demonstrate the presence of subclinical, widespread enlarged RPE cells present in all subjects imaged. In the fovea, the last area to be affected in choroideremia, we found greater disruption to the RPE than to either the photoreceptor or choriocapillaris layers. The unexpected finding of patches of photoreceptors that were fluorescently-labeled, but structurally and functionally normal, suggests that the RPE blood barrier function may be altered in choroideremia. Finally, we introduce a strategy for detecting enlarged cells using conventional ophthalmic imaging instrumentation. These findings establish that there is subclinical polymegathism of RPE cells in choroideremia.

## Abstract (web summary)

The in vivo pathogenesis of the blinding retinal degeneration choroideremia is visualized, which reveals enlarged and area-disrupted retinal pigment epithelial cells, providing evidence for polymegathism in choroideremia.

## Introduction

Choroideremia^1^ is a rare X-linked retinal degeneration caused by loss-of-function mutations in the *CHM* gene that results in nyctalopia, progressive visual field loss, and ultimately, blindness^2–4^. The name of the disease suggests primary involvement of the choroid^5^, a vascularized support structure for the outer retina. For example, choriocapillaris degeneration has been linked with declining visual function in choroideremia^6^. However, several studies have suggested that choroideremia primarily affects the retinal pigment epithelial (RPE) cells^7–11^. Other studies report photoreceptor degeneration as a driver of the disease^12,13^ or that there are multiple interacting factors^14–19^. Given the various factors proposed, and the possibility of discrepancy between cell and animal models compared to the actual clinical manifestation of choroideremia, visualization of the in vivo pathogenesis of the disease across the outer retinal layers is of critical importance for developing future treatments for these patients.

Over two dozen clinical trials are currently underway for choroideremia, many involving gene augmentation^20–23^. A common aim is the preservation of central vision provided by the macular photoreceptors, which often remain intact until the late stages of disease, typically around the fifth decade of life^2^. Although there are numerous structural (e.g., optical coherence tomography (OCT), autofluorescence) and functional (e.g., visual fields, electroretinography) measures for assessing disease progression or treatment efficacy, the majority of these approaches are better suited for assessing photoreceptor structure/function than RPE status. Clinically available techniques (e.g., OCT, autofluorescence) provide information about whether the RPE monolayer is intact, but to date, these methods are unable to provide cellular-level information about the RPE. In the current study we sought to evaluate the RPE layer, along with the photoreceptor and choriocapillaris layer to see to what extent each layer is affected in choroideremia, and to help inform future clinical trials.

Female carriers of choroideremia are typically regarded to be mostly unaffected. Although it is recognized that there is a wide spectrum of disease severity affecting female carriers, severe symptoms such as night blindness and visual impairment similar to those observed in affected males are thought to affect only a minority of the female carriers^24^. In a previous study, abnormal full-field electroretinography responses were detected in only 15% of female carriers, despite 96% of these carriers presenting with ophthalmoscopic signs such as patchy atrophy and coarse granularity in the mid and far periphery^25^. It has been hypothesized that the degree to which female carriers are affected depends on lyonization (random X-inactivation)^12,16,26,27^ within the RPE mosaic, which would result in patches of both mutant and normal RPE cells in which the mutant RPE cells could degenerate over the course of five or more decades of life. In particular, lyonization could explain the patchy pigmentary changes such as melanin clumping due to pigment migration or areas of RPE atrophy reported in female carriers^7,12,28–30^. To our knowledge, this theory has not been conclusively demonstrated to date due in part to the lack of tools for imaging the RPE at a cellular level in patients over large enough areas within the eye to detect mutant and normal patches of RPE.

Adaptive optics (AO) retinal imaging^31^ has provided cellular-level resolution of the living human retina in patients with choroideremia, particularly for assessing the status of cone photoreceptors^6,9,32,33^. Structurally, well-preserved cone photoreceptors have been reported at the margins of RPE atrophy^16,33^ but areas of reduced cone density elsewhere have also been observed^9,16,32^. Functionally, retinal sensitivity assessments showed a sharp drop-off in cone function near areas of atrophy consistent with the sharp loss of cone photoreceptors at or near the atrophic edge^6,33^. In female carriers of choroideremia, cones seem to be relatively well-preserved^32^, but with the possibility of patchy loss of photoreceptors in some eyes^16^. Although these studies have suggested that RPE damage precedes cone photoreceptor degeneration, limited assessment of the RPE mosaic at the cellular level has been performed. Recently, adaptive optics enhanced indocyanine green (AO-ICG) imaging has been introduced as a method for visualizing the RPE mosaic^34^ which has revealed the presence of enlarged RPE cells in Bietti Crystalline Dystrophy^35^. We have also shown that AO-ICG can be used to visualize the choriocapillaris^36^. AO-ICG in combination with other modalities, particularly adaptive optics optical coherence tomography (AO-OCT), provides a means to visualize the RPE mosaic^37–39^. Taken together, these techniques enable in vivo multimodal assessment of the photoreceptor, RPE, choriocapillaris complex at the cellular level in both affected males and female carriers of choroideremia.

## Results

### RPE cells are enlarged in both affected males and female carriers

**Fig. 1:**
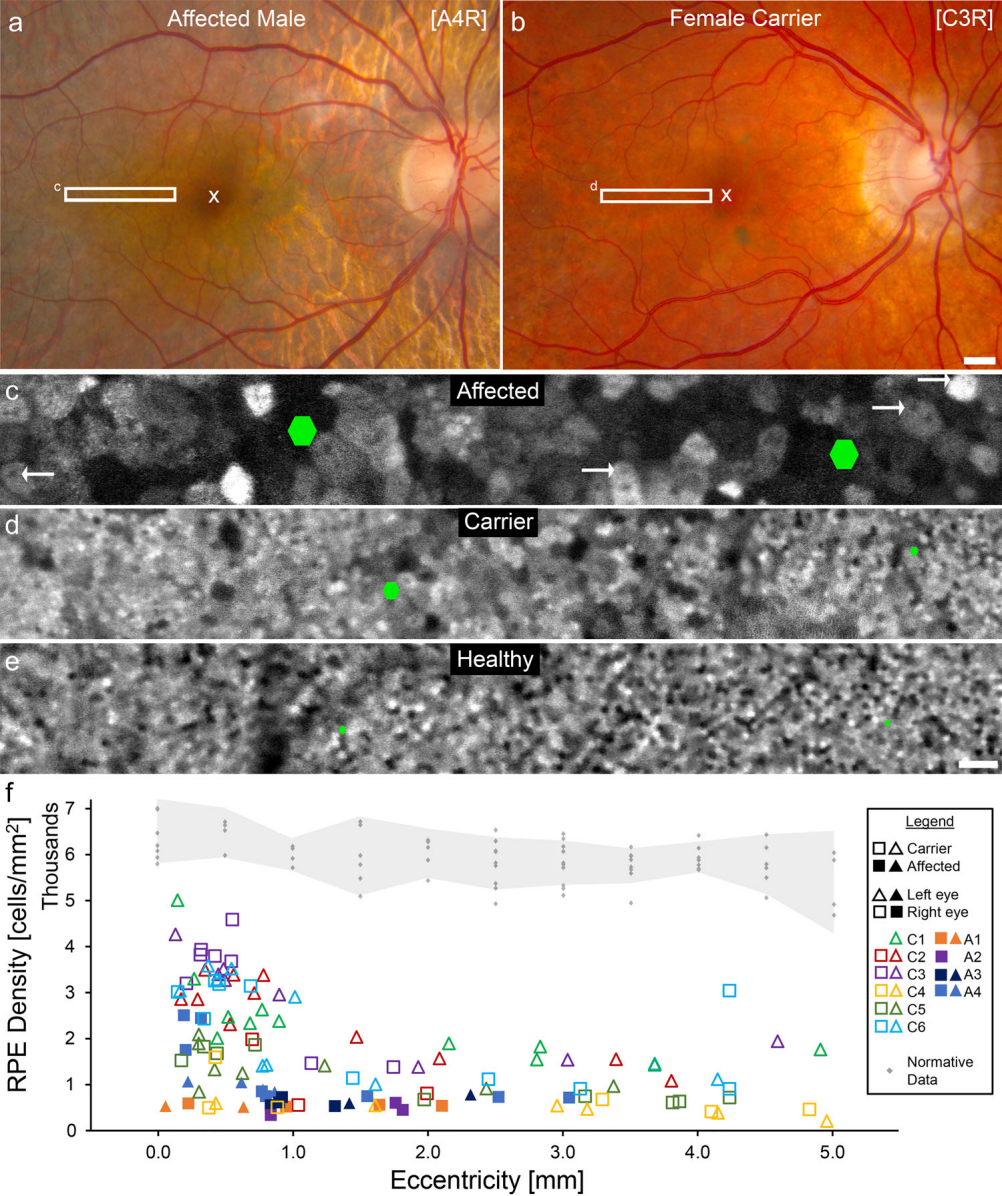
Enlarged retinal pigment epithelial (RPE) cells observed in choroideremia. **a**, **b** Color fundus images of an affected male and a female carrier showing areas of outer retinal atrophy and mild pigmentary changes, respectively. The white ‘x’ denotes the fovea (eccentricity = 0.0 mm). Subject codes are shown in brackets (L = left eye, R = right eye; A = affected male, C = female carrier). The number in the brackets refers to the subject number (Supplementary Table 1). White rectangles indicate areas where high resolution adaptive optics (AO) images were obtained. Scale bar, 1 mm. **c**, **d** Late phase adaptive optics enhanced indocyanine green (AO-ICG) images of the RPE mosaic obtained in the white rectangles from (**a** and **b**). **e** For comparison, an AO-ICG image obtained from a similar area in a healthy right eye is shown. Green hexagons denote the approximate size of single RPE cells in the immediate vicinity of the hexagon (barely visible in the healthy eyes). The nuclei of RPE cells are visible in some of the enlarged cells (arrows point to examples of nuclei visible as hypofluorescent spots). In the healthy eye, RPE cell size is nearly constant across the image; in contrast, the RPE cells in the carrier eye are variable in size, with many of the cells enlarged. The RPE cells in the affected eye are consistently enlarged across the entire image. Scale bar for (**c**–**e**), 100 µm. **f** Quantitative measurements of RPE cell density obtained at different locations (eccentricities) from the fovea out to ~5.0 mm in the temporal direction (female carriers, unfilled symbols; affected males, filled symbols; triangles, left eye; squares, right eye). Measurements of RPE cell density in the affected eyes were possible only up to the edge of atrophy, which was generally <3.0 mm. RPE density in all subjects was significantly lower than expected normal values (gray dots; the gray band represents 99.9% confidence interval)^40^.

The characteristic heterogeneous pattern of fluorescence^34,35^ was observed in all affected males and female carriers imaged using AO-ICG. However, unlike the mosaic of healthy eyes, in choroideremia eyes, the RPE cells were substantially enlarged by up to ~5Χ, especially in the affected males (Fig. 1). In many of the enlarged cells, hypofluorescent RPE nuclei could be observed (arrows, Fig. 1c), consistent with our previous findings revealing nuclei in some larger RPE cells^35^. Evidence of these enlarged RPE cells could not be detected based on fundus autofluorescence or clinical OCT. The AO-ICG fluorescence pattern was observed across the retina in female carriers as well as within the areas where RPE was intact in affected males. Measurements of RPE density were below the 99.9% confidence interval for expected normal RPE density^40^ at all measured eccentricities for both affected males (filled symbols) and female carriers (unfilled symbols). Measurements in affected males were performed up to the edge of atrophy, generally <3 mm (Fig. 1f). Across all eccentricities, the average RPE density in the female carriers (1981 cells/mm^2^) was in between that of healthy eyes (5947 cells/mm^2^) and affected males (844 cells/mm^2^), but there was considerable variability in the female carriers, especially near the foveal center (eccentricity 0.0 mm). For the female carriers, the presence of interspersed enlarged RPE cells among less affected RPE cells (Fig. 1d) is consistent with the hypothesis that there are patches of mutant RPE cells distributed throughout areas with normal RPE cells in which the X-chromosome that harbors the loss-of-function mutation has been inactivated during the lyonization process.

**Fig. 2:**
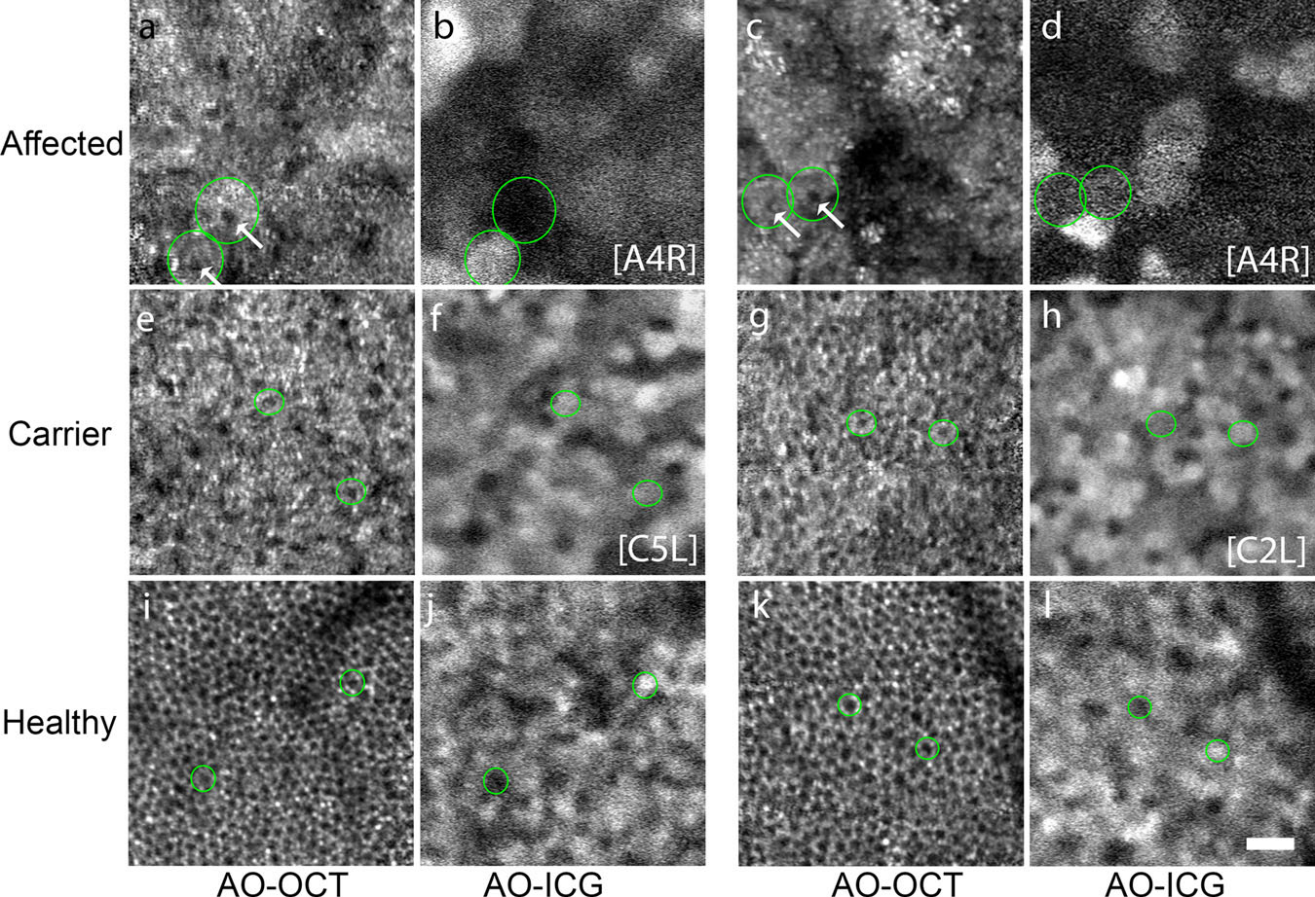
Multimodal imaging corroborates the finding that the RPE cells are enlarged in choroideremia. Co-registered adaptive optics optical coherence tomography (AO-OCT) images and (AO-ICG) images from choroideremia are shown in comparison to healthy examples. **a**–**h** RPE cells from both affected males and female carriers are enlarged compared to (**i–l**) healthy RPE cells. Green circles are examples of individual RPE cells from each pair of images. RPE cell nuclei can be seen as the small dark spots in AO-OCT images (e.g., white arrows). The increased spacing between cell nuclei in choroideremia compared to healthy eyes can be seen in the AO-OCT images. The retinal locations (eccentricities) of the images are (**a**, **b**) 1.0 mm, (**c**, **d**) 1.5 mm, (**e**–**h**) 0.5 mm, (**i**, **j**) 0.5 mm, and (**k**, **l**) 1.5 mm. Scale bar, 50 µm.

Multimodal AO imaging incorporating co-registered AO-OCT and AO-ICG images also showed that RPE cells were enlarged in choroideremia (Fig. 2). For a subset of subjects (three affected males and five female carriers), AO-OCT images of RPE cells were also obtained to evaluate whether RPE cells could be detected. Although AO-OCT imaging in healthy eyes has been successfully demonstrated, to our knowledge, there are limited examples of AO-OCT imaging performed in patients with advanced disease, which can be challenging due to the longer acquisition time needed to obtain averaged volumes for RPE cell imaging (125–150 volumes per location; see *Methods: Adaptive Optics Imaging*). As previously demonstrated, side-by-side comparison of the same RPE cells imaged using AO-OCT and AO-ICG helps to establish how to interpret images of disrupted RPE^37^, which can appear strikingly different compared to healthy RPE. Whereas RPE cells can be identified on the basis of differences in overall fluorescence intensity in AO-ICG images^41^, in AO-OCT images, the darker RPE cell centers can be used to infer cell-to-cell spacing, most easily visible when the RPE cells are in close proximity to each other. In healthy subjects (Fig. 2i, k) the cell centers are tightly packed and in female carriers (Fig. 2e, g) they are sparsely distributed. In the affected male, it is difficult to distinguish the boundaries of individual RPE cells in the AO-OCT images, but comparison of the AO-ICG images alongside AO-OCT images suggests that the dark spots correspond to cell centers within enlarged RPE cells (Fig. 2a–d). In general, the sources of image contrast are not yet well understood when the cellular structure itself is as dramatically altered as it is in the case of these enlarged RPE cells, which introduces challenges for image interpretation. Nonetheless, these multimodal AO images provide an initial glimpse into the characteristics of AO-OCT RPE imaging in diseased eyes and help to confirm that the RPE monolayer is intact even in areas of low cell contrast (AO-OCT shows the presence of RPE cells in hypofluorescent areas of the AO-ICG image, and conversely, AO-ICG shows the presence of RPE cells in low-contrast areas of the AO-OCT image). Taken together, these data suggest the possibility for substantial RPE enlargement to occur in the setting of an intact RPE monolayer as revealed by clinical OCT scans. However, whether this RPE disruption occurs concomitant with photoreceptor or choriocapillaris degeneration remains to be shown.

### RPE cells are disrupted to a greater extent than surrounding neural and vascular layers

**Fig. 3:**
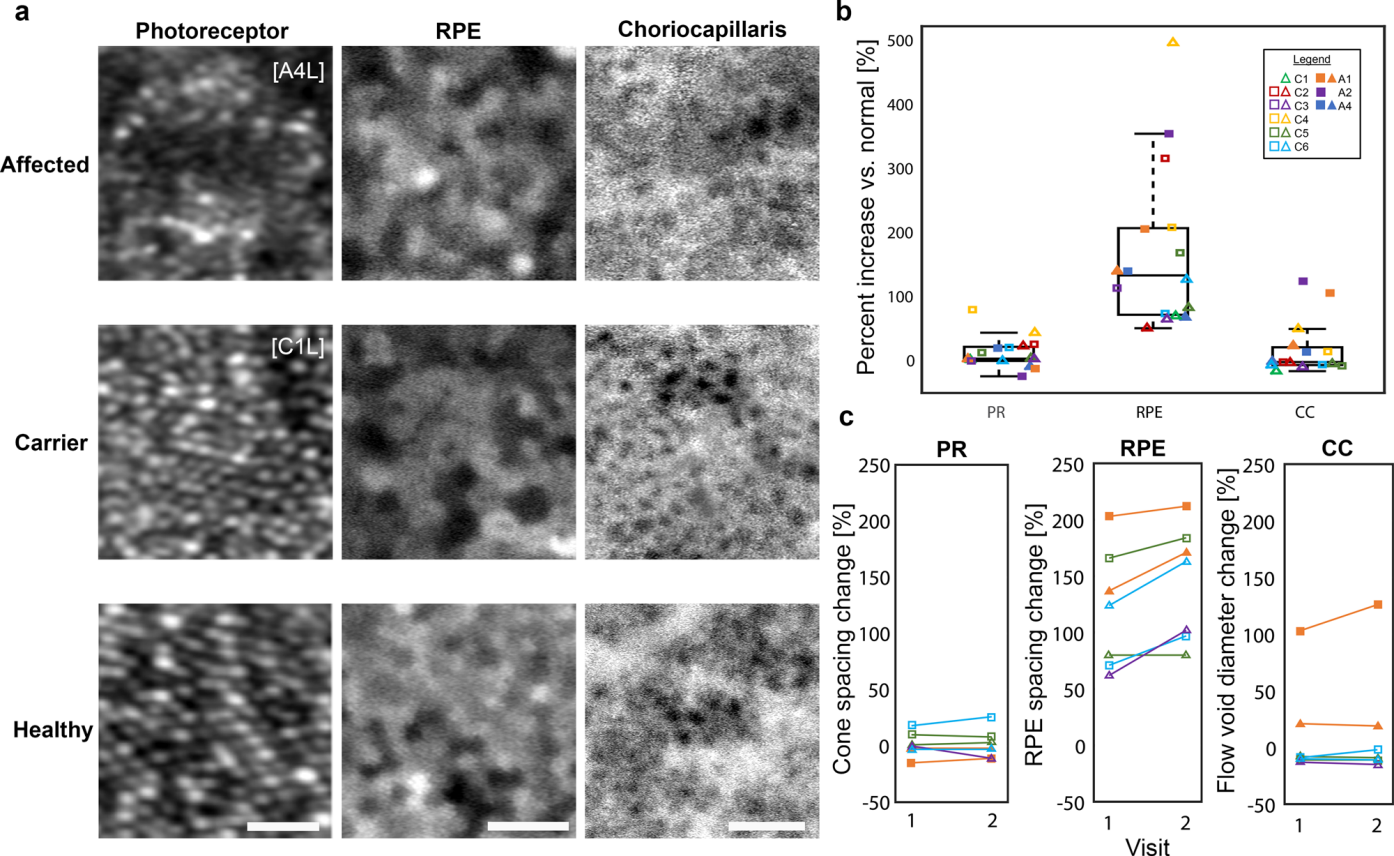
Visualization of the photoreceptor, RPE, choriocapillaris complex in choroideremia. **a** AO images acquired at the fovea (eccentricity = 0.0 mm) showing foveal cone photoreceptors (PR), RPE cells, and the choriocapillaris (CC) microvasculature. Images from an affected male, a female carrier, and a healthy eye are shown. Note that subject A4L has a relatively well-preserved island of RPE cells at the fovea in which RPE cells are less enlarged compared to other affected males; outside of the fovea, RPE cells still form a contiguous monolayer but are dramatically enlarged (see Fig. 5a). Scale bars: PR, 10 µm; RPE, 50 µm; CC, 100 µm**. b** Box plots of photoreceptor spacing, RPE spacing, and choriocapillaris flow void diameters show that the RPE is the most severely affected layer of these three layers (center line: median; box limits: upper/lower quartiles; whiskers: 1.5× interquartile range; points beyond the whiskers: outliers). Data corresponding to the subjects shown in (**a**) can be determined using the legend. Measurements of photoreceptors, RPE, and choriocapillaris performed in choroideremia were compared to normative histologic data^43^, normative in vivo RPE data^40^, and normative in vivo choriocapillaris data^36^, respectively. For subjects who had two visits, only data from the first visit was used for this analysis. **c** Longitudinal imaging acquired at the same location and co-registered across visits revealed the degree to which photoreceptors, RPE, and choriocapillaris changed from one visit to the next (time between visits varied between 2 and 12 months; Supplementary Table 1). The largest changes were observed in the RPE layer, further corroborating our finding that the RPE layer is the most disrupted layer out of these three layers.

We performed AO-ICG imaging to visualize the photoreceptor, RPE, choriocapillaris complex in vivo in 5 eyes from 3 affected males and 11 eyes from 6 female carriers (Fig. 3). Currently, AO-ICG imaging of the choriocapillaris can only be generated during the transit phase immediately after dye injection which limits the imaging of the choriocapillaris to a small field of view (0.6 mm × 0.6 mm). As central vision is typically well-preserved until the late stages of the disease, we opted to acquire the choriocapillaris images at the foveal center of each subject. At this location, the photoreceptor, RPE, choriocapillaris complex was intact in all subjects imaged and the relative size scale of each layer was quantified (foveal cone spacing, RPE cell spacing, and choriocapillaris flow void effective diameter)^36^. The difference in size scale compared to normal was dramatically increased in the RPE layer (average cone spacing increase compared to the expected normal value, 9% [25%]; RPE spacing increase, 165% [125%]; choriocapillaris flow void diameter increase, 15% [43%]; mean [SD]) (Fig. 3b). The degree to which the RPE was enlarged was significantly increased compared to both the photoreceptor (*P* < 0.001) and choriocapillaris (*P* < 0.001), but there was no significant difference between the photoreceptors and choriocapillaris (*P* = 0.63). In general, these observations applied to both affected males and female carriers.

To further explore whether there were differences in the rate of progression of each of the three layers, longitudinal imaging of the same region was performed in a subset of subjects who were able to return for a second visit (Supplementary Table 1). In this subset of 2 eyes from 1 affected male and 5 eyes from 3 female carriers, the visit-to-visit change was greatest in the RPE layer, followed by the choriocapillaris, and then the photoreceptors (average foveal cone spacing, RPE cell spacing, and choriocapillaris flow void effective diameter increase from visit 1 to visit 2: cone photoreceptors, 1% [6%]; RPE, 24% [15%]; choriocapillaris, 4% [9%]; mean [SD]) (Fig. 3c). The visit-to-visit increase in size scale observed in the RPE was significantly greater than those observed in both the photoreceptor (*P* < 0.01) and choriocapillaris (*P* < 0.05) layers; there was no significant difference between the photoreceptors and choriocapillaris (*P* = 0.49). These findings suggest a faster rate of progression in the RPE compared to the surrounding layers and is consistent with our overall observation that the RPE is severely disrupted in choroideremia. Interestingly, in two of the carrier eyes (C3L and C5R), the RPE mosaic (AO-ICG fluorescence pattern) appears to shift slightly from visit to visit above the more stable choriocapillaris even over a relatively short follow-up period of 2–6 months (Supplementary Movie 1), which contrasts with the stable mosaic observed in healthy subjects across years^35^. This might be due to the dropout of individual RPE cells within and near the imaging field of view, resulting in a local, compensatory rearrangement of cells to preserve the continuity of the RPE monolayer.

### Disruption of outer retinal barrier function of the RPE

**Fig. 4:**
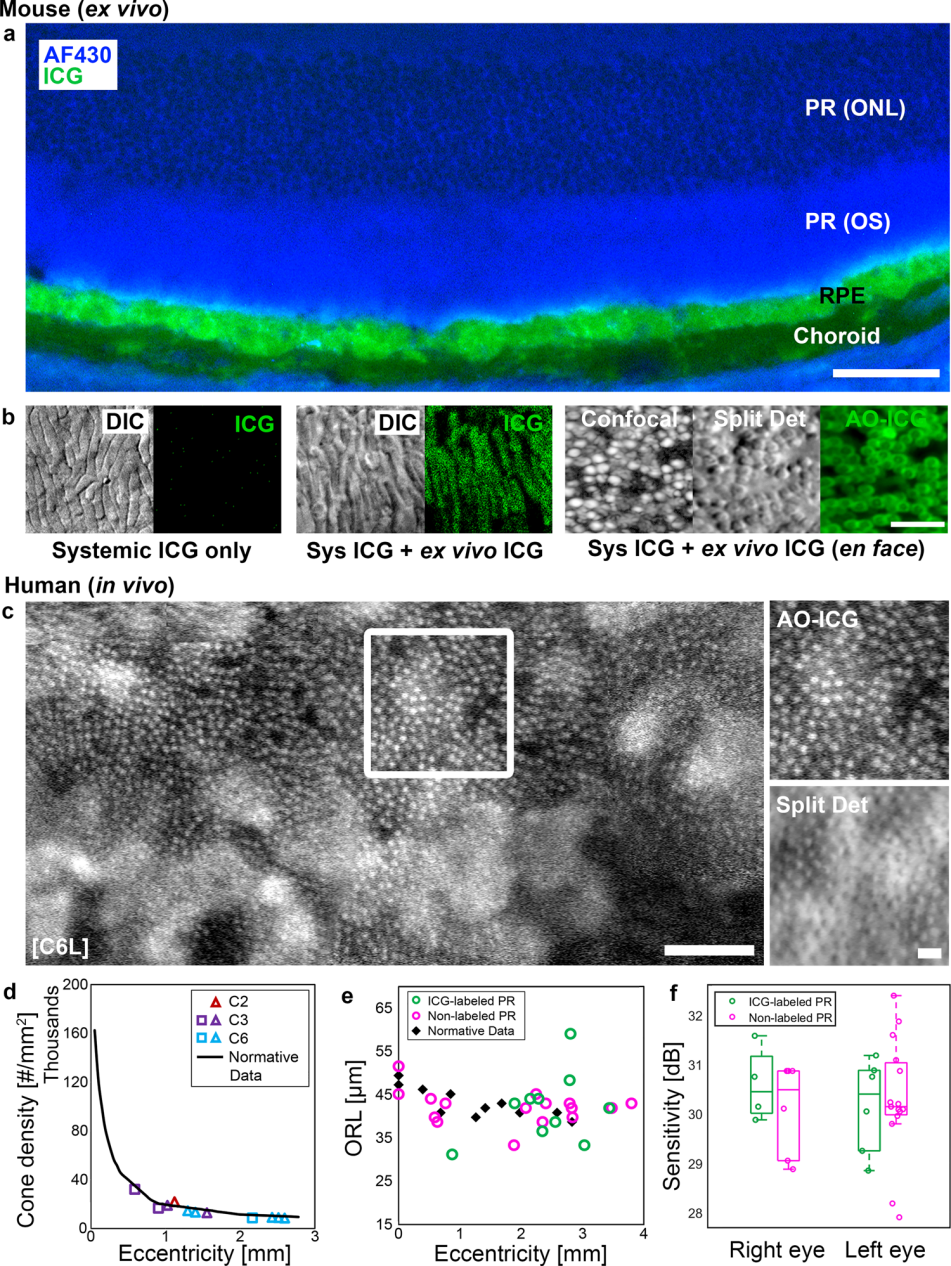
ICG labeled photoreceptors in mice and human. **a** Following intraperitoneal injection of ICG in mice, ICG can be detected within the RPE in unfixed cryosections, as has been previously demonstrated^34^. The overlying photoreceptors (PR) remain unlabeled under normal conditions due to the tight junction between RPE cells, which together establish the outer blood retinal barrier. The PR outer segments (OS) and outer nuclear layer (ONL) can be discerned using autofluorescence imaging (430 nm). A faint infrared autofluorescence can be observed in the choroid due to melanin. Scale bar, 50 µm. **b** No detectable ICG signal was observed in the photoreceptor layer after systemic injection of ICG. The presence of photoreceptor profiles was confirmed using differential interference contrast (DIC) microscopy. However, following an ex vivo incubation with ICG, the photoreceptors were readily labeled with ICG. These results were further corroborated using a custom-assembled adaptive optics microscope capable of simultaneous acquisition of *en face* confocal reflectance (displayed in log scale), non-confocal split detection, and AO-ICG images of the photoreceptor mosaic. Scale bar (all images in **b**), 10 µm. **c** ICG labeled photoreceptors observed in a female carrier. The heterogeneous RPE fluorescence pattern can be observed in the background. Zooms of the white box show that the fluorescently labeled photoreceptors are consistent with photoreceptors imaged using non-confocal split detection. Scale bars: 50 µm (C6L larger image), 10 µm (zooms, AO-ICG and Split Det). **d** The densities of cone photoreceptors in areas of ICG labeling are similar to normative histologic values^43^. **e** Outer retinal length (ORL) measurements were performed using AO-OCT images acquired in areas of ICG labeling and in areas without ICG labeling. There was no apparent difference in ORL between labeled or non-labeled photoreceptors, and both were similar to ORL measurements performed in healthy eyes. **f** Retinal sensitivity measurements (microperimetry) performed within patches of ICG labeled photoreceptors were within normal limits and similar to measurements obtained in neighboring, non-labeled photoreceptors.

Patches of fluorescently labeled photoreceptors were observed in choroideremia. To further characterize this phenomenon, we modeled the uptake of ICG dye into the RPE cells using mice. Building upon our previous studies showing that the murine RPE layer is selectively labeled following intraperitoneal administration of ICG^34,35^, we performed additional experiments to examine the status of the photoreceptors in mice (Fig. 4). Here, we confirmed that the overlying photoreceptors were not labeled with ICG under normal physiologic conditions (Fig. 4a). The presence of ICG within RPE confirmed that the dye was successfully delivered to the mouse eye. Even though the photoreceptors and RPE cells are in close apposition with each other, after carefully separating the retina from the RPE layers through dissection, we confirmed that the photoreceptors remained unlabeled after systemic administration of ICG (Fig. 4b). However, photoreceptors were readily labeled with ICG following ex vivo incubation of the separated retina with ICG, suggesting that the tight junctions between RPE cells which comprise the outer blood retinal barrier effectively prevent the photoreceptors from being labeled with ICG under normal conditions. We also repeated this condition with imaging performed using *en face* AO microscopy^42^ (same excitation and emission spectra used for human AO-ICG imaging), further corroborating this finding. These results demonstrate that photoreceptors are readily labeled when exposed to ICG.

Upon close examination of the AO-ICG images in human participants, patches of fluorescently labeled photoreceptors were observed in 10 eyes from 5 female carriers as well as in 1 eye from 1 affected male (Fig. 4c and Supplementary Fig. 1). A total of 11 distinct patches of ICG labeled cone photoreceptors were selected from the female carriers for further analysis. All ICG labeled cones identified in the AO-ICG images were colocalized with cones identified in simultaneously acquired, co-registered non-confocal split detection images (*n* = 2317 cones), supporting our claim that these fluorescently labeled structures are indeed cone photoreceptors. Although we did detect a small subset of non-labeled cones (392 cones visible on non-confocal split detection but not on AO-ICG), we found most of the cones within these patches were labeled (86%). The presence of fluorescently labeled cones overlying hypofluorescent RPE cells suggests that this finding is not due to the cone imprinting phenomenon^34^. Quantification of cone density of the cone mosaic within these patches (including both fluorescently labeled and non-labeled cones) yielded values comparable to normative histologic data (Fig. 4d)^43^. In addition, from the AO-OCT images, measurements of outer retinal length (ORL) were performed, defined as the distance between the inner segment/outer segment junction (ellipsoid zone) and the RPE bands^44^. ORL measurements in both labeled and non-labeled photoreceptor regions from five female carriers further showed no statistically significant differences between these two groups (*P* = 0.45). Overall, the ORL in female carriers were similar to normative data from healthy eyes. Finally, fundus-guided retinal sensitivity measurements performed during the same visit as AO-ICG in 2 eyes from 1 female carrier revealed that both ICG labeled and non-ICG labeled photoreceptors had normal retinal sensitivity values (normal range: 29–31 dB)^45^. There were no significant differences in retinal sensitivity observed between labeled and non-labeled patches (ICG labeled, 30.3 ± 0.9 dB; non-labeled 30.2 ± 1.1 dB, *P* = 0.81; *n* = 10 ICG labeled and 21 non-labeled patches, mean ± SD). Altogether, these results suggest that ICG labeled cone photoreceptors in choroideremia are neither structurally nor functionally deficient (i.e., the labeling of photoreceptors with ICG is a result of disruption to the blood barrier function of the RPE and does not represent a defect in the photoreceptors themselves).

It is possible that these patches arise transiently during the initial remodeling of the RPE layer following focal dropout of neighboring RPE cells, that eventually resolve following re-establishment of tight junctions between remodeled RPE cells. Unfortunately, it was difficult to assess RPE cells underlying patches of fluorescently labeled photoreceptors as the fluorescent signal from the photoreceptors obscured the RPE mosaic (Supplementary Fig. 1). However, in one patient, simultaneously-acquired darkfield images of the RPE mosaic^46^ were visible in which there appeared to be RPE enlargement underlying a patch of fluorescently-labeled photoreceptors (Supplementary Fig. 2). This degree of RPE enlargement cannot be explained by eccentricity-dependent differences in RPE morphology^40^, which would support the theory that these patches arise due to defects in the RPE. Further examination of OCT scans and fundus autofluorescence in patches of fluorescently labeled photoreceptors revealed that half (50%) of the patches were associated with a neighboring vitelliform-like lesion. RPE underlying vitelliform lesions are expected to be hypocyanescent due to blocking of the ICG fluorescence^47^. These lesions appeared to be of adult onset when comparisons were made with previous multimodal imaging from the same patients. We therefore hypothesize that these vitelliform-like lesions are secondary to the underlying retinal degeneration (choroideremia) and are a further indication of RPE dysfunction.

Although we observed many examples of patches of ICG labeled photoreceptors, especially in female carriers (Supplementary Figs. 1 and 3), collectively, these still represent a very small portion of the overall retinal area imaged (examples of areas without fluorescently-labeled photoreceptors can be seen in Supplementary Fig. 4). The relatively uniform labeling of photoreceptors in patches overlying even hypofluorescent RPE cells is similar to the ICG labeled photoreceptors that we labeled ex vivo in mice, suggesting that high resolution ICG imaging could lead to insights about the integrity of the outer blood retinal barrier function of the RPE.

### Enlarged RPE cells can be detected using late phase ICG imaging even without adaptive optics

**Fig. 5:**
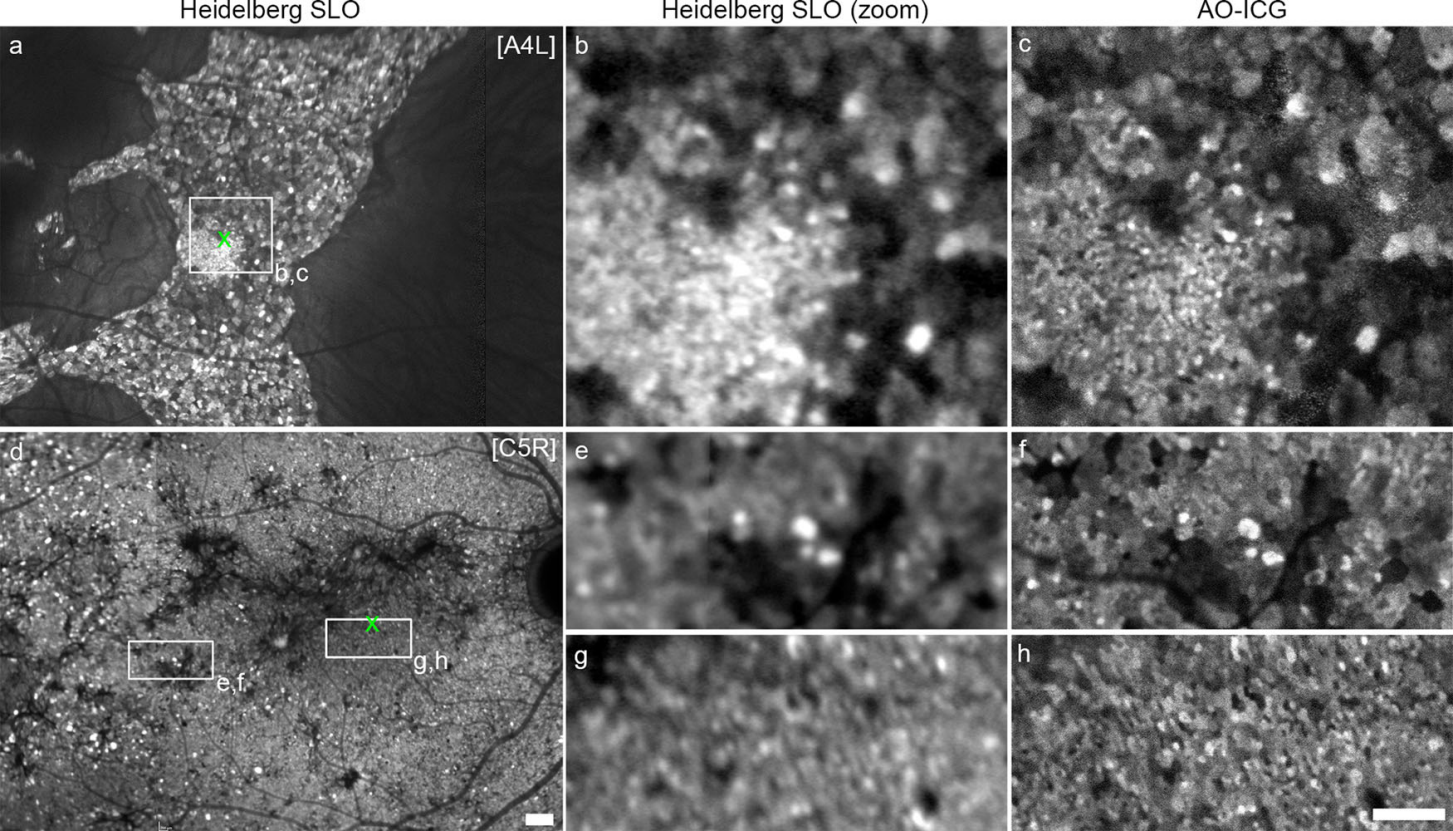
Detection of RPE cell polymegathism using late phase ICG imaging. **a** Late phase ICG image acquired using a commercially available Heidelberg scanning laser ophthalmoscope (SLO) showing widespread enlarged RPE cells in an affected male. **b** Zoom of the white square in (**a**), contrast adjusted to show cellular detail. **c** AO-ICG image of the same region as (**b**). Many of the cells observed in (**c**) can be detected in (**b**). In the lower-left corner of (**b** and **c**), the RPE cells are smaller than those in the upper-right corner, but individual RPE cells are still visible. **d** Heidelberg SLO image from a female carrier. Numerous hyperfluorescent enlarged RPE cells can be observed, especially near the left edges of the image. **e**, **g** Contrast-adjusted zoom of white rectangles. **f**, **h** AO-ICG images corresponding to (**e** and **g**) showing that the heterogeneous pattern of fluorescence captured using the Heidelberg SLO matches the pattern imaged using AO-ICG. These side-by-side comparisons illustrate that individual enlarged RPE cells can be detected using conventional imaging, even without AO. **a**, **d** The green ‘x’ denotes the fovea. Scale bar, 1 mm. **b**, **c**, **e**–**h** Scale bar, 500 µm.

Alongside AO-ICG imaging, late phase ICG imaging using a commercially available scanning laser ophthalmoscope (SLO) (Spectralis, Heidelberg Engineering) revealed that individual enlarged RPE cells could be identified even without the use of AO (see Fig. 5 and Supplementary Movie 2 for a visualization of how the heterogeneous pattern of fluorescence develops in the time between the mid-late and “true” late phases). Although ICG imaging is routinely performed in clinical practice, it is not common to use it to capture the late phase (in this study, defined as 45 min or longer after intravenous injection). While one affected male (subject A4) had a smaller island of better-preserved RPE cells within the larger island of intact RPE cells, the other three affected males had uniformly enlarged RPE cells throughout the entire remaining retina, which could be readily determined using late phase ICG, but not mid-late phase ICG (Supplementary Movie 2). Identifying such patients might be critical for assessing the results of possible therapeutic interventions.

AO imaging typically uses smaller imaging fields of view (<0.6 mm × 0.6 mm) and is more time- and resource-intensive than current clinical testing. Hence, the ability to quickly capture a snapshot of the heterogeneous RPE pattern with an SLO across a wider field of view (9 mm × 9 mm) could be of value for choroideremia, and in particular for the female carriers (Fig. 5d and Supplementary Figs. 3–5). In one female carrier (subject C4), the dramatically enlarged RPE cells were similar in appearance between female carriers and affected males (Supplementary Fig. 5), who had RPE density measurements similar to those of affected males (Fig. 1f). Across all female carriers, small, scattered hyperfluorescent spots were observed using conventional imaging. We confirmed that these corresponded to enlarged RPE cells seen in AO-ICG images obtained in overlapping areas. Likewise, in addition to the hyperfluorescent enlarged RPE cells, larger than expected patches of hypofluorescent RPE cells can also be observed. Together these findings illustrate the extent to which carrier retinas are affected and provide a rare glimpse into the pattern of lyonization in an adult eye. Considering the relatively long time-course of this disease (~decades), and the possibility of maintaining well-preserved photoreceptors despite enlarged RPE cells (e.g., affected males), longitudinal monitoring of the late phase ICG signal (Supplementary Movie 1) in larger cohorts of female carriers may be justified for understanding how mutant colonies of RPE cells give rise to the milder pigmentary changes reported for female carriers.

In summary, the comparisons between conventional SLO and AO-ICG images suggest the possibility for routine assessment of the RPE mosaic at the cellular level in choroideremia and other diseases affecting the RPE, a potentially transformative tool that can be readily implemented into clinical practices without the need for new or complex AO instrumentation.

## Discussion

Our data are in agreement with the view that choroideremia is primarily an RPE disease^10,33^ revealing enlarged RPE cells (Fig. 1) and increased distance between RPE cell centers (Fig. 2). These disruptions to the otherwise contiguous RPE monolayer (as assessed using fundus autofluorescence and OCT) occur earlier than clinically evident. To our knowledge, this is the first in vivo evidence of polymegathism of the RPE cells in choroideremia, and it is similar to the RPE polymegathism reported in age related macular degeneration^48–50^. Some of the enlarged RPE cells may be multinucleated (e.g., the two arrows on the far right of Fig. 1c and arrows in Supplementary Fig. 4), which has also been suggested as a protective mechanism for the RPE to help maintain homeostasis^51^.

Whereas progressive RPE changes were observed over a period as short as 2 months, the cone photoreceptors and choriocapillaris at the fovea remain relatively stable during the same period. Although there may be differences between preservation of these layers in the fovea vs. at eccentric locations, our observation of normal structure and function even in fluorescently labeled photoreceptors are in agreement with previous studies showing well-preserved cone photoreceptor structure up to^16,33^ or beyond the edge of RPE atrophy^6^, as well as preserved retinal sensitivity^6,33^. However, as we are unable to assess the state of rod photoreceptors from our imaging, it is unclear when and to what degree rods are affected relative to the cones, RPE, and choriocapillaris. Rods have also been reported to be a primary site of degeneration in choroideremia^12^ and future studies comparing rods and RPE cells in vivo are warranted.

Despite the relatively milder disease observed clinically in female carriers, our imaging clearly shows widespread enlarged RPE cells present in these patients. Our data are consistent with histological findings of polymegathism of the RPE observed in a female carrier^52^. In a subset of the female carriers, we also explored whether disease severity was related to skewed X-inactivation. Here, we evaluated X-inactivation ratio in five affected heterozygous females by digest analysis at the AR and RP2 loci on peripheral blood-derived DNA. Segregation analysis was possible in three female carriers who had a male sample to determine the expression of the pathogenic *CHM* allele (Supplementary Table 2). Fundus autofluorescence imaging was used to assess disease severity. Four female carriers were considered to have mild disease, and two intermediate; for the affected males, two were considered intermediate, two severe, and one mild (Supplementary Table 1). There was no clear relationship between disease severity and X-inactivation. Nonetheless, the scattered hyper- and hypo-fluorescent regions visible in the late phase ICG images (Supplementary Figs. 3–5) provide a glimpse into the in vivo lyonization pattern within the RPE mosaic that ultimately serve as loci of RPE degeneration within these eyes. This late phase ICG pattern of patchy cyanescence appears to be a characteristic pattern, observed in 100% of the female carriers imaged.

The observation of ICG labeled photoreceptors in the majority of female carriers imaged is notable, as it suggests a defect in RPE integrity. Even though most of the fluorescently labeled photoreceptors were found on female carriers (mild or intermediate severity), we did find patches of these photoreceptors in one affected male (subject A4, intermediate severity) (Supplementary Fig. 1). However, since these cone photoreceptors appeared to have both normal structure and function, we concluded that this phenomenon likely represents a defect in the RPE. The fact that some of these patches of labeled photoreceptors appeared in different locations at different times (2–6 months) in the subset of female carriers that underwent longitudinal imaging, also supports the hypothesis that subclinical remodeling of RPE cells occurs within the intact monolayer and can be revealed using late phase ICG.

Fluorescence ICG imaging is routinely used for visualizing the integrity of retinal and choroidal vasculature. Here, we show that late-phase ICG imaging is also valuable for visualizing enlarged RPE cells in patients using ophthalmoscopes with or without AO. AO-ICG imaging, however, provides higher resolution and cellular detail, but it is inherently more time and resource intensive; a larger montage constructed from approximately one hundred overlapping AO images (Supplementary Figs. 3–5) requires ~30–45 min to acquire (not including the waiting time after injection). In contrast, acquisition of conventional late phase ICG images that cover a much larger area of the retina requires only a few minutes using conventional SLO. We also demonstrated that SLO imaging is particularly useful for identifying areas where the RPE mosaic is disrupted in female carriers, who have patches of enlarged RPE cells distributed throughout the retina. For affected males, these images reveal the extent to which the remaining RPE is affected and may be particularly relevant for selecting candidate patients for gene therapy or other therapeutic interventions, especially if they have an area of better-preserved RPE within the larger preserved area (Fig. 5a). Our findings also help to explain the smooth and mottled pattern of autofluorescence that has been previously reported in some affected males^53^. This non-angiographic application using ICG is already available in many clinics and can be readily translated to clinical practice.

Overall, affected males showed substantial RPE disruption across their remaining retina, but our data suggests that female carriers should also be considered as part of the continuum of disease. The patches of enlarged RPE cells within female carriers might be useful as an in vivo model for studying the early stages of RPE disruption in affected males. Moreover, when viewed at the level of individual RPE cells, the commonality and similarity of having clusters of enlarged RPE cells suggests a common disease mechanism between the affected males and female carriers. These findings warrant increased efforts toward developing treatments for symptomatic female carriers as well as exploring the use of RPE-based endpoint measures monitoring treatment efficacy in affected males^20–23,54^. We expect that visualization of the RPE at the cellular level will have a positive impact on current and future gene therapy trials, as a means to better inform patients of their status and eligibility for treatment, to monitor possible risks as treatment trials progress, and also as a potential outcome of clinical evaluations for choroideremia^55^. Ultimately, including female carriers who exhibit signs of RPE disease alongside affected males in such trials^24^ could help to accelerate progress toward realizing a cure for the disease.

In conclusion, there is subclinical, widespread polymegathism of RPE cells in choroideremia, present in both affected males and female carriers. Progressive RPE changes are observed in the fovea in choroideremia, but the overlying photoreceptors and underlying choriocapillaris remain intact and relatively stable. We demonstrate the possibility of detecting enlarged RPE cells using commercially available, conventional ophthalmic imaging in combination with late phase ICG. This approach may be useful as a clinical measure for tracking the progression of choroideremia or for monitoring the integrity of the outer blood retinal barrier function of the RPE in this blinding retinal disease.

## Methods

### Clinical evaluation of patients

Six female carriers and five affected males of choroideremia were recruited from the National Eye Institute eye clinic for this study (NCT02317328; https://clinicaltrials.gov). All patients had molecularly confirmed pathogenic or likely pathogenic variants in *CHM*, and no known allergies to shellfish, iodine, or ICG. Participants who were willing to return for a follow-up visit were invited for follow-up adaptive optics (AO) imaging. All patients underwent best corrected visual acuity testing, dilated funduscopic examination, multimodal AO imaging, ocular biometry (IOL Master, Carl Zeiss Meditec), color fundus photography (Topcon), OCT (Spectralis, Heidelberg Engineering), and autofluorescence imaging (Spectralis, Heidelberg Engineering). Autofluorescence images relative to the patient’s age were used to determine disease severity^24^. ICG was only administered in patients 18 years and older. Mid-late and late phase ICG images were acquired (Spectralis, Heidelberg Engineering), where mid-late phase was defined as 15–30 min after administration of the first dose of ICG and late phase as >45 min.

### Adaptive optics imaging

Eyes were dilated using 2.5% phenylephrine hydrochloride (Akorn Inc) and 1% tropicamide (Sandoz, A Novartis Division). A custom-assembled multimodal AO instrument incorporating confocal reflectance^56^, non-confocal split detection imaging^57^, AO-ICG^34–36^ and AO-OCT^37^ was used for imaging. AO-OCT and AO-ICG imaging were performed sequentially. During imaging, subjects were advised to blink naturally, and frequent breaks were taken between video acquisitions. Prior to each imaging session, light power levels were measured at the corneal plane and maintained <135 µW for the 790 nm light source (reflectance and AO-ICG), <45 µW for 880 nm (wavefront sensing), and <1.45 mW for 1080 nm (AO-OCT).

For AO-ICG imaging (performed in 6 female carriers and 4 affected males), a field of view ranging from 1.5 to 2 degrees (750 × 605 pixels) was used. Confocal reflectance and non-confocal split detection images were simultaneously recorded alongside AO-ICG. Imaging was performed in three parts: before, during, and after i.v. administration of ICG at a dose of 25 mg distributed in two doses (1 + 2 mL). In part 1 (~10–15 min per eye, including breaks), reflectance images were acquired at overlapping retinal locations between the fovea and ~5.0 mm temporal (female carriers) or up to the atrophic border (affected males). Simultaneously-captured AO-ICG images confirmed the absence of ICG signal. In part 2 (~5–10 min per eye), the fovea of each eye was imaged for the first few minutes after administration of each ICG dose. The second dose (2 mL) was administered 10 min after the first dose (1 mL). In part 3 (~30–45 min per eye), a larger area covering the fovea, parafovea, and a temporal strip of retina was imaged. For AO-ICG, part 3 imaging was typically performed 30–45 min after the initial injection of dose 1^34,35^. In total, ~50–150 video locations were acquired per eye, depending on the amount of imaging time available. Videos were acquired at a speed of 17 frames per second (~9 s videos were used for parts 1 and 3; for part 2, a 2 min video was used, followed by a 45 s video). Following acquisition, AO-ICG images from parts 1 and 3 were processed to correct for eye motion, and then overlapping images were assembled into montages using Photoshop (Adobe, San Jose, CA, USA)^34,58^. Images of the choriocapillaris were obtained from part 2 and co-registered to the images from parts 1 and 3^36^.

For AO-OCT imaging (performed in 5 female carriers and 3 affected males), a 1.5 degree field of view (300 × 300 pixels) was used. Repeated AO-OCT volumes were collected in order to reveal the RPE^37–39^. For photoreceptor imaging, 50 volumes were obtained for each retinal location and for RPE imaging, 125–150 volumes were obtained for each retinal location. To ensure sufficient time for decorrelation of the speckle pattern observed in the RPE layer between video acquisitions, retinal locations were paired, and acquisition was alternated between the two locations (acquisition of 5 volumes at a time per location). The spectral domain AO-OCT system had a speed of 147 kHz which enabled volumes to be acquired at a speed of 1.6 volumes per second. In total, ~450 volumes were acquired per eye (~20–30 min). Following acquisition, retinal volumes were digitally flattened based on the outer retinal layers, and then a 2D en face projection of the photoreceptor layers^59^ was used to compute eye motion correction in the lateral direction, following the same registration procedures used for AO-ICG images^58^. RPE images were extracted from a single en face slice of the averaged OCT volume.

The retinal scaling factor for conversion from degrees to mm was computed using a paraxial ray trace on a three-surfaced simplified model eye^60^ updated with the subject’s biometry information obtained after dilation (axial length, corneal curvature, and anterior chamber depth)^61^.

### RPE density measurements

Using AO-ICG images, 11 eyes from 6 female carriers and 7 eyes from 4 affected males were used for RPE quantification. For each eye, regions of interest (ROIs) were selected from the fovea (0 mm) out to 5 mm eccentricity in the temporal direction (female carriers) or up to the atrophic border (affected males). For each ROI, RPE cells were manually identified by an expert grader. A second expert grader reviewed all ROIs and performed additional manual correction, flagged for additional review by the first expert grader. This iterative process continued until either full agreement between the two graders was reached, or the image was discarded. Out of a total of 125 ROIs selected, 7 containing large areas of hypofluorescence that made grading difficult were discarded (6% of the ROIs). The identified cell centers were used to construct Voronoi diagrams from which cell densities were calculated. Any Voronoi neighborhood that exceeded the boundary of the ROI was discarded and the number of remaining cells was divided by the total area of the remaining Voronoi neighborhoods.

### Photoreceptor, RPE, and choriocapillaris measurements

For the cross-sectional analysis, 11 eyes from 6 female carriers and 5 eyes from 3 affected males were evaluated (one subject was excluded due to unstable fixation during the part 2 AO-ICG imaging). Longitudinal evaluation was performed in 5 eyes from 3 female carriers and 2 eyes from 1 affected male. For each eye, a staircase of ROIs was selected^36^: a 300 µm square ROI was selected from the choriocapillaris image, followed by a 200 µm square ROI from an AO-ICG image of RPE, selected within the larger choriocapillaris ROI; finally, a 50 µm square ROI was selected from a confocal reflectance image of photoreceptors, selected within the RPE ROI. Cones and RPE cells were manually identified by two expert graders and iterative correction to the markings were performed until mutual agreement was reached. Similarly, flow voids were manually segmented from the choriocapillaris image by two expert graders until consensus was reached. Cell spacing was quantified based on the density recovery profile^62,63^ and the average effective diameter of choriocapillaris flow voids (diameter of the circle with equivalent area) was quantified^36^.

### Quantification of ICG labeled cones using AO-ICG

In select image regions where ICG labeled photoreceptors were observed, correspondence between photoreceptors in AO-ICG and non-confocal split detection images were qualitatively assessed, and cell centers were manually identified separately for each modality by an expert grader. Two additional expert graders reviewed all ROIs and performed additional manual correction until full agreement between the three graders was achieved. The identified cell centers were used to construct Voronoi diagrams from which cell densities were calculated.

### Outer retinal length measurements

ORL was measured in AO-OCT as the distance between the inner segment/outer segment junction (ellipsoid zone) and the RPE bands^44^.

### Retinal sensitivity measurements

Retinal sensitivity measurements were performed on 2 eyes from 1 female carrier (subject C4) 2 days after AO-ICG imaging. Immediately after AO-ICG imaging was completed on the first day, AO-ICG images were processed and montaged to determine locations at which ICG labeled photoreceptors were present. Test points located above ICG labeled photoreceptors and control points above non-labeled photoreceptors (within ~1° of test points) were determined based on the AO-ICG imaging data and coordinates transferred to a color fundus photo. Fundus-controlled perimetry was performed using a COMPASS device (iCare, Finland) controlled with an external laptop using the open perimetry interface (OPI)^64^. Specifically, we built an interactive web application in the software environment *R*, with the add-on libraries Shiny and OPI^64,65^. This web application allowed us to acquire an infrared reflection (IR) reference image with the COMPASS device. A color fundus photograph (with labeled test points) was then registered to this IR reference image using the affine transformation from the ‘Landmark Correspondences’ function in ImageJ. The coordinates of all test and control points (in terms of the COMPASS IR reference image) were then extracted, converted from pixels to degrees, and transferred to the web application. At each test point, a Goldmann III (0.43° diameter, ~126 μm in an emmetropic eye) stimulus was presented against a photopic background (10 cd/m^2^)^66^. Threshold at each test point was obtained using a Bayesian ZEST threshold strategy with a uniform prior and a stop criterion of SD < 0.5 dB for the probability density function^67^. For the given test points (average eccentricity for OD of 9.62° [8.48°, 11.2°] and for OS of 8.67° [6.85°, 9.9°]), a sensitivity of 29–31 dB can be considered as normal considering the age of the carrier^45^.

### Mouse histology

All mice were housed in the NIH animal facilities. Two female mice (C57BL/6 J, 3-month-old; BALB/cJ, 4-month-old) were used for this study to replicate previously-published procedures described in pigmented^34^ and albino^35^ mice. Intraperitoneal injection of ICG (200 µL of 5 mg/mL) was performed. 16 h later, mice were euthanized by CO_2_ inhalation and then eyes were enucleated. For the pigmented mouse, immediately after enucleation, the eye was embedded in optical cutting temperature compound, rapidly frozen using acetone (cooled to approximately −70 °C by addition of dry ice), and then cryosectioned through the center of the eye (8–10 µm thick). A single cryosection was collected on a Super-frost Plus microscope slide (Fisher Scientific), vacuum dried, and mounted in Immu-Mount (ThermoFisher Scientific) immediately before imaging. For the albino mouse, after enucleation, the eye was hemisected using McPherson-Vannas scissors 14124 (World Precision Instruments) while immersed in media (Gibco FluroBrite DMEM, ThermoFisher Scientific) cooled to ~0 °C by ice surrounding the dissection chamber. After removing the lens and anterior segment, the neural retina was gently detached from the RPE with fine forceps and divided into four equal pieces. One piece served as a control. The other pieces were incubated with 2 µM ICG dissolved in media for 1 h (37 °C, 5% CO_2_), and then washed five times with the Gibco FluroBrite DMEM (ThermoFisher Scientific) media.

For the pigmented sample, microscopy was performed using an Axio Imager.Z2 slide scanning epifluorescence microscope (Zeiss) equipped with a 20X/0.8 Plan-Apochromat (Phase-2) non-immersion objective (Zeiss), a high resolution ORCA-Flash4.0 sCMOS digital camera (Hamamatsu), a 200 W X-Cite 200DC broad band lamp source (Excelitas Technologies), and 10 customized filter sets (Semrock) optimized to detect the following fluorophores: DAPI, Alexa Fluor 405, Alexa Fluor 430, Alexa Fluor 488, Alexa Fluor 546, Alexa Fluor 594, Alexa Fluor 647, IRDye 680LT, IRDye 800CW, and PerCP, with all filters specially configured to minimize spectral crosstalk for 10-color epifluorescence imaging^68^. Alexa Fluor 430 filter set was used for imaging autofluorescence and IRDye 800CW filter set for detection of the specific ICG fluorescence signal.

For the albino sample, microscopy was performed using either a custom-modified confocal microscope (SP8, Leica) or a custom assembled AO microscope^42^. For imaging using the SP8, samples were transferred to glass slides and covered with a 0.17 mm thick coverslip with the photoreceptor layer facing the coverslip. Silicone grease was applied surrounding the sample to control the gap between the slide and the coverslip, in addition to anchoring the coverslip. A high NA objective (HC PL APO CS2 40×/1.30 OIL, Leica) was used. 3D differential interference contrast (DIC) and ICG fluorescence images were sequentially acquired. DIC images were acquired using a 488 nm CW laser diode and a transmitted light photomultiplier tube detector. ICG images were excited using a 730 nm CW laser diode and detected using an avalanche photodiode with an 810/90 nm bandpass filter. For imaging using the custom assembled AO microscope, we placed the sample in the chamber that was anchored by a mesh instead of the glass coverslip to avoid the reflection of the light on the glass-water interface. This microscope was constructed using Thorlabs CERNA components and a Nikon 0.8 NA water-dipping objective coupled with an AO scanning light ophthalmoscope outfitted with confocal reflectance^56^, non-confocal split detection^57^, and AO-ICG^34–36^ capabilities.

### X-chromosome inactivation assay

The X-chromosome Inactivation (XCI) assay was performed using the human *RP2* GAAA repeat primers and the human *AR* CAG repeat internal primers reported previously^69^. Briefly, the HpaII (NEB) digested and undigested DNA samples were amplified using the RP2 and AR primers using the NEBNext Ultra II Q5 master mix (NEB) in a multiplex reaction, in which the forward primers were FAM-labeled. The PCR products were then mixed with a size standard and analyzed on an ABI 3500 automated genetic analyzer (Applied Biosystems). The peak areas were called using the GeneMapper software (Applied Biosystems) and the X-inactivation for each alleles was calculated using this formula: (d1/u1)/[(d1/u1) + (d2/u2)], where d1 and d2 refer to the peak areas of allele 1 and allele 2 in the digested samples, and u1 and u2 refer to those of allele 1 and allele 2 of the undigested samples, respectively. The XCI assay was performed on samples from five heterozygous females, of which segregation analysis was performed using a sample from a first-degree affected male relative to determine the extent of inactivation of the variant allele.

### Study approval

For animals, experiments were conducted according to protocols approved by the NIH IACUC. For human subjects, research procedures adhered to the tenets of the Declaration of Helsinki. The study was approved by Institutional Review Board of the National Institutes of Health (NCT02317328). Written, informed consent was obtained from all patients after the nature of the research and possible consequences of the study were explained.

### Statistics and reproducibility

Two-tailed unpooled *t*-tests were used to compare groups. For the pair-wise comparisons between three groups (e.g., photoreceptor, RPE, and choriocapillaris layer), the Bonferroni correction for multiple tests was applied. Statistically significant values are displayed as (*P* < 0.001), (*P* < 0.01), and (*P* < 0.05). For non-significant differences the corresponding “*P* = ” are shown in the methods section. The data used to perform the *t*-test are displayed as mean [SD] or mean ± SD. For Fig. 1f, the 99.9% confidence interval is displayed as a gray band. For Fig. 3b, box plots are displayed for the three groups (center line: median; box limits: upper/lower quartiles; whiskers: 1.5× interquartile range; points beyond the whiskers: outliers). The individual data points and sample sizes corresponding to the plots in the figures and used in the statistical analysis are provided in Supplementary Data. Quantitative image analysis was carried out with multiple expert graders (for more details, please refer to the “RPE Density Measurements”, “Photoreceptor, RPE, and Choriocapillaris Measurements”, and “Quantification of ICG labeled cones using AO-ICG” sections above).

### Reporting summary

Further information on research design is available in the Nature Research Reporting Summary linked to this article.

## Supplementary information

Peer Review File

Supplementary Information

Description of Additional Supplementary Files

Supplementary Movie 1

Supplementary Movie 2

Supplementary Data 1

Reporting Summary

## Data Availability

All data generated or analyzed during this study are included in this published paper (and its supplementary information and supplementary data files). Source data is located in Supplementary Data 1.
