# Supplementary material for: Widespread subclinical cellular changes revealed across a neural-epithelial-vascular complex in choroideremia using adaptive optics
Source: Commun Biol. 2022 Sep 13;5:893. doi: 10.1038/s42003-022-03842-7 (PMC9470576; doi:10.1038/s42003-022-03842-7)
Supplement: Supplementary file 2 — Supplementary Information [file 42003_2022_3842_MOESM2_ESM.pdf]

Supplementary Information

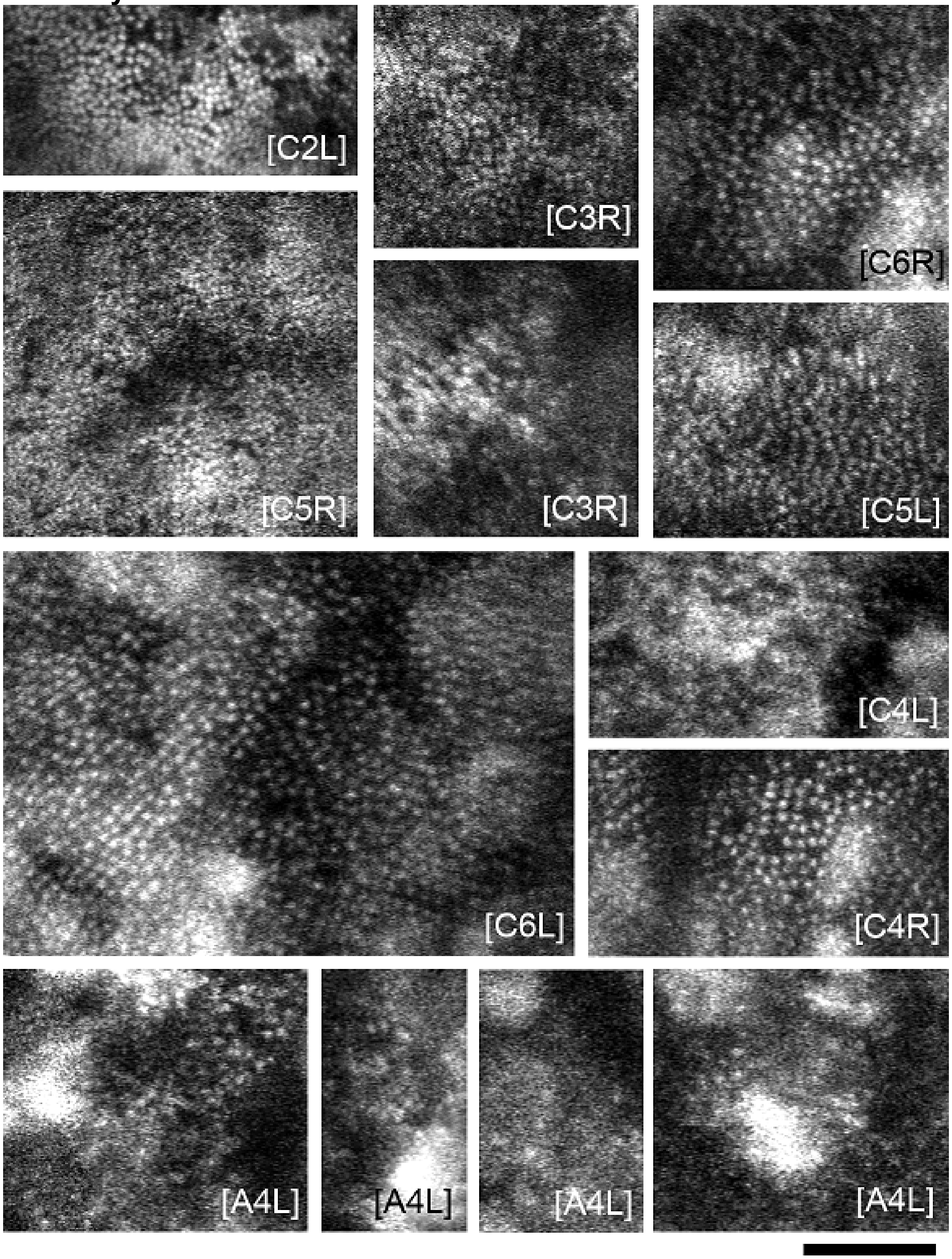

Supplementary Figure 1. Additional examples of ICG labeled photoreceptors observed in choroideremia. Patches of ICG labeled photoreceptors were observed. The heterogeneous pattern of ICG fluorescence from the RPE can be observed in the background. Scale bar, 100 μm.

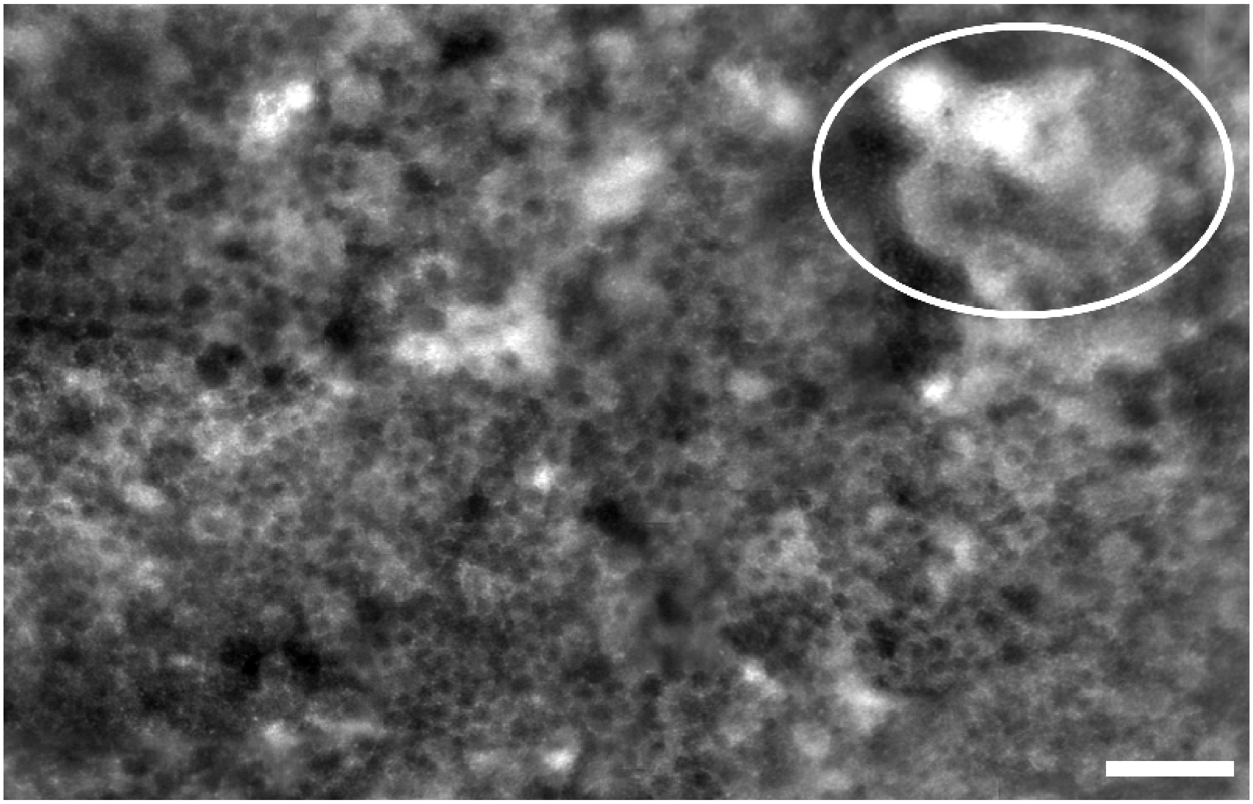

Supplemetary Figure 2. Darkfield RPE imaging in Subject C2L. There is a patch of fluorescently labeled photoreceptors overlying the circled area. The pattern of RPE cells appears to be enlarged within the circled area compared to the surrounding regions of the image. There is no vitelliform-like lesion neighboring this patch. Scale bar, 100 μm.

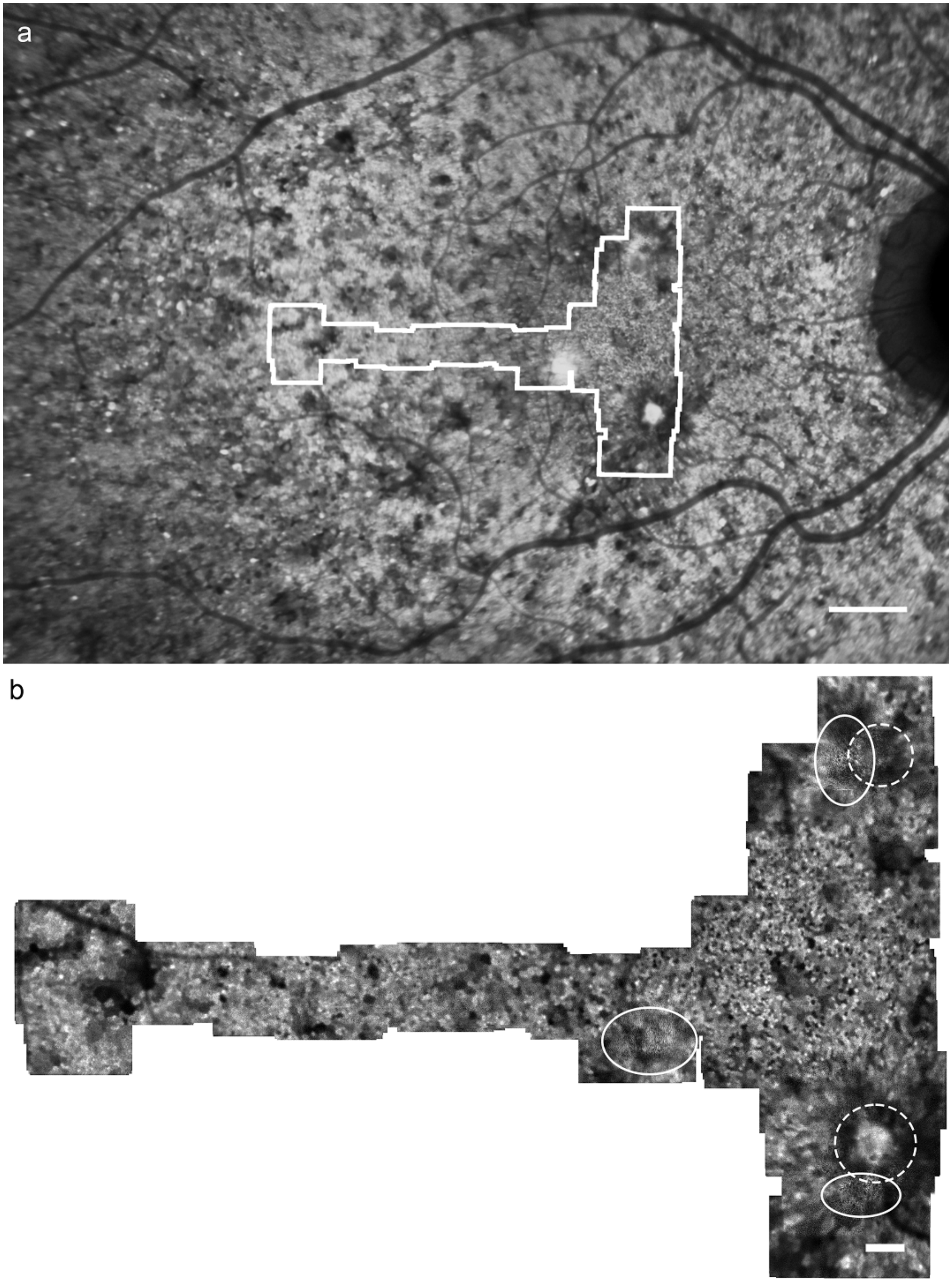

Supplementary Figure 3. Late phase ICG image of female carrier (subject C3R). Acquired using **(a)** SLO (Spectralis, Heidelberg) and **(b)** AO-ICG (zoom of outline in a). Solid circles contain patches of ICG labeled photoreceptors. Dotted circle is a small vitelliform lesion. Scale bars, a: 100 mm, b: 200 µm.

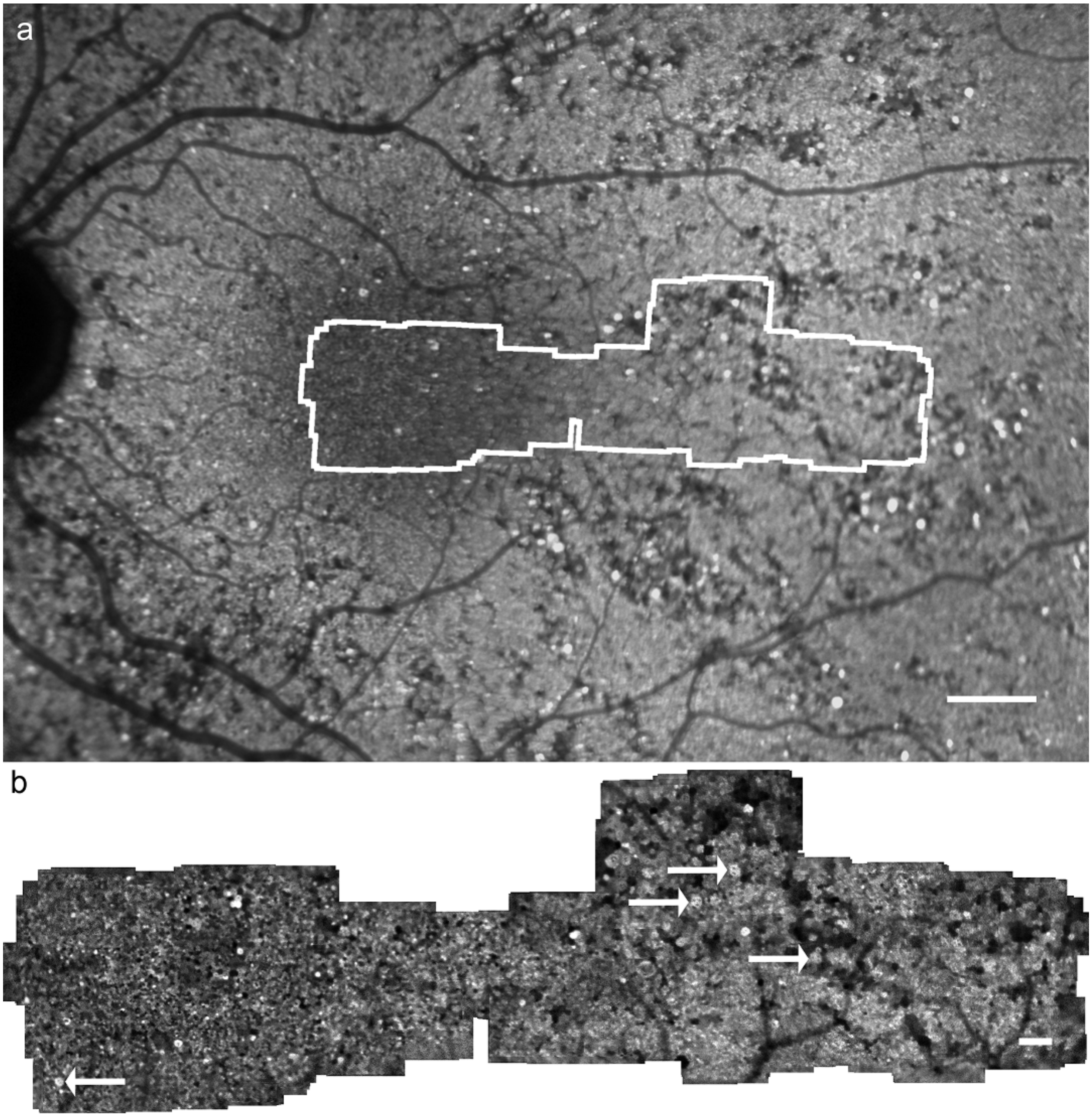

Supplementary Figure 4. Late phase ICG image of female carrier (subject C1L). Acquired using **(a)** SLO (Spectralis, Heidelberg) and **(b)** AO-ICG (zoom of outline in a). Fluorescently labeled cone photoreceptors were not observed in the area imaged in b. Arrows are examples of possible multinucleated enlarged RPE cells. Scale bars, a: 100 mm, b: 200 µm.

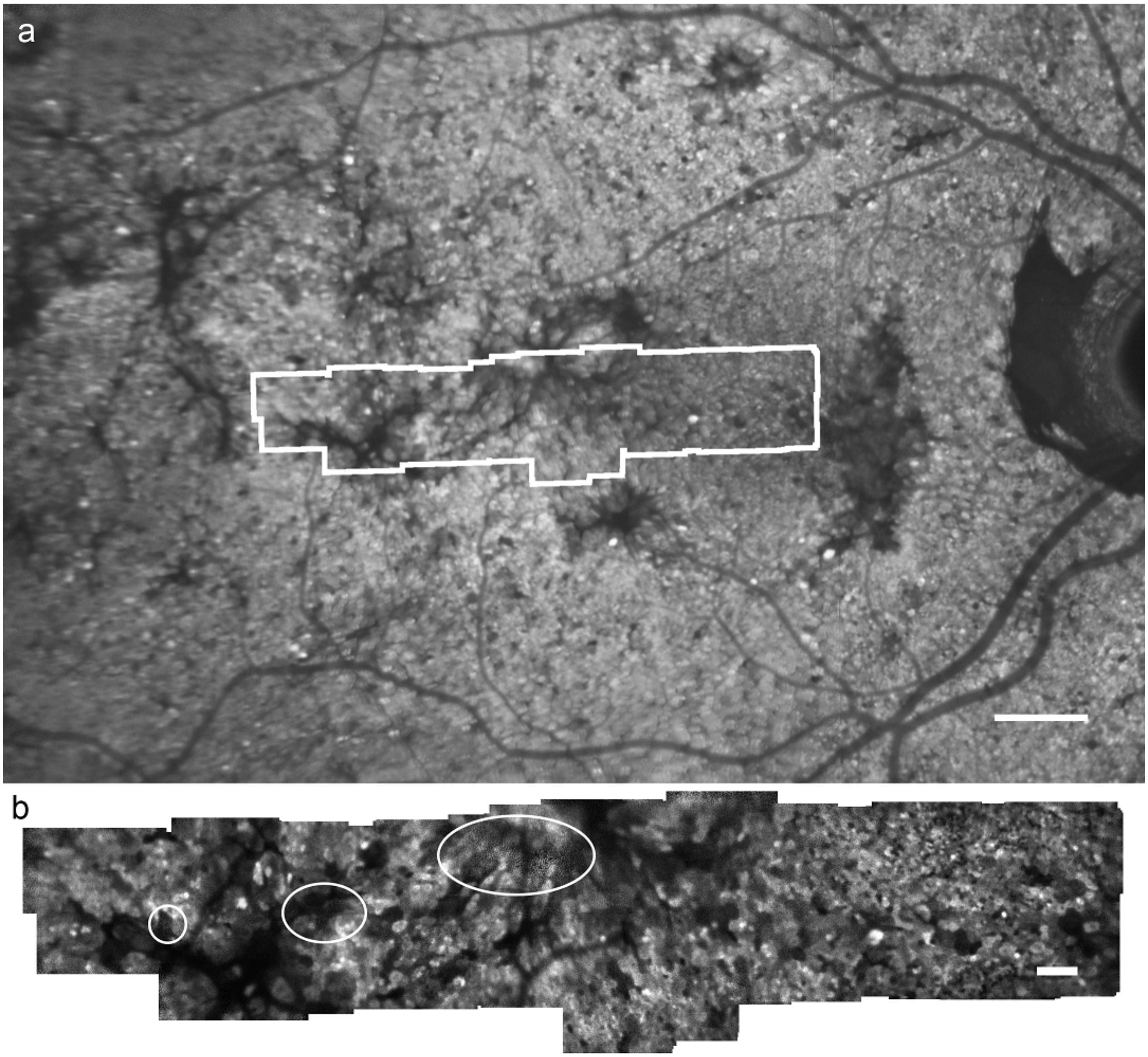

Supplemental Figure 5. Late phase ICG image of female carrier (subject C4R). Acquired using **(a)** SLO (Spectralis, Heidelberg) and **(b)** AO-ICG (zoom of outline in a). Solid circles contain patches of ICG labeled photoreceptors. Scale bars, a: 100 mm, b: 200 μm.

Supplementary Table 1. Subject information

ID[1]	Visits[2]	Age[3]	BCVA[4]	Severity[5]	*CHM* variant (NM_00390.2)
C1 (F1)	1	21	20/32<br>20/20	Mild	Exon 15 deletion, heterozygous
C2 (F3)	1	35	20/20<br>20/16	Intermediate	c.525_526delAG:p.(Glu177LysfsTer6), heterozygous
C3 (F4)	2 [+6]	37	20/20<br>20/20	Mild	c.37delG:p.(Val13Ter), heterozygous
C4 (F7)	1	49	20/20<br>20/20	Intermediate	c.757C>T:p.(Arg253Ter), heterozygous
C5 (F1)	2 [+2]	50	20/20<br>20/10	Mild	Exon 15 deletion, heterozygous
C6 (F2)	2 [+4]	52	20/20<br>20/12.5	Mild	c.1157_1160del:p.(Cys386Serfs*22), heterozygous
A1 (F1)	2 [+12]	18	20/32<br>20/20	Intermediate	Exon 15 deletion, hemizygous
A2 (F6)	1	35	20/32<br>20/25	Severe	c.225G>A:p.(Trp75Ter), hemizygous
A3 (F5)	1	37	20/50<br>20/63	Severe	c.49+2dupT, hemizygous
A4 (F1)	1	42	20/16<br>20/16	Intermediate	Exon 15 deletion, hemizygous
A5 (F2)[6]	1	15	20/16<br>20/16	Mild	c.1157_1160del:p.(Cys386Serfs*22), hemizygous

[1] Subject ID: C = female carrier, A = affected male. The family (F) # is given in parenthesis. Note that all subjects are Caucasian.
[2] For subjects with 2 visits, the duration between visits, in months, is given in brackets.
[3] Age at first visit.
[4] Best corrected visual acuity; right eye, followed by left eye.
[5] Severity was assessed based on fundus autofluorescence images.
[6] ICG imaging was only performed in subjects who were 18 years of age or older. Hence, subject A5 did not undergo ICG imaging. However, this subject was used to help interpret genetic testing results from subject C6, including the X-chromosome Inactivation assay.

Supplementary Table 2. X-inactivation

		AR marker		RP2 marker		Mean of AR/RP2 Xi (%)
Subject ID	Severity	Fragment lengths (bp)	XCI patten (%)	Fragment lengths (bp)	XCI patten (%)	
C2	Intermediate	235/256	46:54	370/390	34:66	60.0
C5	Mild	223/244	29:71	370/378	44:56	63.5
C6	Mild	226/238	65:35	370/374	65:35	65.0
C1	Mild	220/244	26:74	370/378	45:55	67.5
C3	Mild	229/241	7:93	370/374	21:79	86.0

The XCI patterns were close between the carriers, except carrier C3 who showed skewed XCI. Since we did not have a male sample for carriers C2 or C3, it was not possible to determine whether the mutant allele is more activated than that of the normal allele.

Supplementary Movie 1 (separate file). Longitudinal imaging of the RPE and choriocapillaris at the fovea. Images from subjects C6L, C6R, C5R, and C3L are shown. The RPE shows changes from visit to visit while choriocapillaris remain stable.

Supplementary Movie 2 (separate file). Mid-late and late phase images of ICG in affected males. Images from subjects A1L, A1R, A2L, A2R, A3L, A3R, A4L, and A4R are shown. Mid-late ICG images were taken 15-30 minutes after injection and late ICG images were taken 45 minutes or more after injection. Choroidal vessels can be seen in mid-late ICG images but not in the late ICG images. The heterogeneous RPE fluorescence pattern emerges in the late ICG images. Widespread enlarged RPE cells are visible in all eyes in the late ICG images.
