# Supplementary material for: Widespread subclinical cellular changes revealed across a neural-epithelial-vascular complex in choroideremia using adaptive optics
Source: Commun Biol. 2022 Sep 13;5:893. doi: 10.1038/s42003-022-03842-7 (PMC9470576; doi:10.1038/s42003-022-03842-7)
Supplement: Supplementary file 3 — Description of Additional Supplementary Files [file 42003_2022_3842_MOESM3_ESM.pdf]

Description of Additional Supplementary Files

File name: Supplementary Movie 1

Description: Longitudinal imaging of the RPE and choriocapillaris at the fovea. Images from subjects C6L, C6R, C5R, and C3L are shown. The RPE shows changes from visit to visit while choriocapillaris remain stable.

File name: Supplementary Movie 2

Description: Mid-late and late phase images of ICG in affected males. Images from subjects A1L, A1R, A2L, A2R, A3L, A3R, A4L, and A4R are shown. Mid-late ICG images were taken 15-30 minutes after injection and late ICG images were taken 45 minutes or more after injection. Choroidal vessels can be seen in mid-late ICG images but not in the late ICG images. The heterogeneous RPE fluorescence pattern emerges in the late ICG images. Widespread enlarged RPE cells are visible in all eyes in the late ICG images.

File name: Supplementary Data 1

Description: Numerical source data used to create Figure 1f, Figure 3b,c, and Figure 4d,e,f in the paper.
