# Supplementary material for: Widespread subclinical cellular changes revealed across a neural-epithelial-vascular complex in choroideremia using adaptive optics
Source: Commun Biol. 2022 Sep 13;5:893. doi: 10.1038/s42003-022-03842-7 (PMC9470576; doi:10.1038/s42003-022-03842-7)
Supplement: Supplementary file 7 — Reporting Summary [file 42003_2022_3842_MOESM7_ESM.pdf]

nature portfolio

Corresponding author(s): Johnny Tam

Last updated by author(s): Jul 28, 2022

Reporting Summary

Nature Portfolio wishes to improve the reproducibility of the work that we publish. This form provides structure for consistency and transparency in reporting. For further information on Nature Portfolio policies, see our Editorial Policies and the Editorial Policy Checklist.

Statistics

For all statistical analyses, confirm that the following items are present in the figure legend, table legend, main text, or Methods section.

n/a	Confirmed	
☐	☒	The exact sample size (*n*) for each experimental group/condition, given as a discrete number and unit of measurement
☐	☒	A statement on whether measurements were taken from distinct samples or whether the same sample was measured repeatedly
☐	☒	The statistical test(s) used AND whether they are one- or two-sided<br>*Only common tests should be described solely by name; describe more complex techniques in the Methods section.*
☒	☐	A description of all covariates tested
☒	☐	A description of any assumptions or corrections, such as tests of normality and adjustment for multiple comparisons
☐	☒	A full description of the statistical parameters including central tendency (e.g. means) or other basic estimates (e.g. regression coefficient) AND variation (e.g. standard deviation) or associated estimates of uncertainty (e.g. confidence intervals)
☐	☒	For null hypothesis testing, the test statistic (e.g. *F*, *t*, *r*) with confidence intervals, effect sizes, degrees of freedom and *P* value noted<br>*Give P values as exact values whenever suitable.*
☒	☐	For Bayesian analysis, information on the choice of priors and Markov chain Monte Carlo settings
☒	☐	For hierarchical and complex designs, identification of the appropriate level for tests and full reporting of outcomes
☒	☐	Estimates of effect sizes (e.g. Cohen's *d*, Pearson's *r*), indicating how they were calculated

Our web collection on statistics for biologists contains articles on many of the points above.

Software and code

Policy information about availability of computer code

Data collection	Data collection was performed using custom software that has all been previously reported. Custom written software was used for the acquisition of data (refs 34-37) and preprocessed according to a previously-reported custom-written algorithm (ref 58) (see methods).
Data analysis	A combination of commercially-available and custom-written software was used for data analysis. A previously-published algorithm that utilizes ImageJ and Matlab was used to generate images of choriocapillaris (ref 36). Adobe Photoshop CC and Microsoft Excel were used for data visualization and statistical analysis.

For manuscripts utilizing custom algorithms or software that are central to the research but not yet described in published literature, software must be made available to editors and reviewers. We strongly encourage code deposition in a community repository (e.g. GitHub). See the Nature Portfolio guidelines for submitting code & software for further information.

Data

Policy information about availability of data

All manuscripts must include a data availability statement. This statement should provide the following information, where applicable:

- Accession codes, unique identifiers, or web links for publicly available datasets
- A description of any restrictions on data availability
- For clinical datasets or third party data, please ensure that the statement adheres to our policy

All data are available in the main text or the supplementary materials.

Field-specific reporting

Please select the one below that is the best fit for your research. If you are not sure, read the appropriate sections before making your selection.

☒ Life sciences ☐ Behavioural & social sciences ☐ Ecological, evolutionary & environmental sciences

For a reference copy of the document with all sections, see nature.com/documents/nr-reporting-summary-flat.pdf

Life sciences study design

All studies must disclose on these points even when the disclosure is negative.

Sample size	As our study presents a new method for visualizing ICG-labeled cells in choroideremia, rather than an attempt to illustrate a statistically significant biological phenomenon, no sample size considerations were relevant. That said, the number of patients included is larger than the typical sample sizes for adaptive optics retinal imaging studies.
Data exclusions	Since adaptive optics video sequences are captured, we excluded any raw data captured during blinks, excessive eye motion, or other drop in image quality that prevented image sequences from being registered to a reference frame, which is the standard approach for adaptive optics retinal imaging data.
Replication	The proposed imaging technique was applied to a cohort of both female carriers and affected males, demonstrating the repeatability of the approach. We verified our observations of RPE disruption was repeatable by performing a longitudinal analysis on four of the patients, during which time the subjects underwent the same procedures. We showed that the finding of substantial RPE disruption was observed across multiple visits.
Randomization	Randomization was not applicable, since the position of each sample was not likely to influence the outcome.
Blinding	Blinding was not applicable, since no anticipated result was expected.

Reporting for specific materials, systems and methods

We require information from authors about some types of materials, experimental systems and methods used in many studies. Here, indicate whether each material, system or method listed is relevant to your study. If you are not sure if a list item applies to your research, read the appropriate section before selecting a response.

Materials & experimental systems

n/a	Involved in the study	
☒	☐	Antibodies
☒	☐	Eukaryotic cell lines
☒	☐	Palaeontology and archaeology
☐	☒	Animals and other organisms
☐	☒	Human research participants
☒	☐	Clinical data
☒	☐	Dual use research of concern

Methods

n/a	Involved in the study	
☒	☐	ChIP-seq
☒	☐	Flow cytometry
☒	☐	MRI-based neuroimaging

Animals and other organisms

Policy information about studies involving animals; ARRIVE guidelines recommended for reporting animal research

Laboratory animals	Two female mice (C57BL/6J; BALB/cJ) were used for this study.
Wild animals	did not involve wild animals
Field-collected samples	did not involve samples collected from the field
Ethics oversight	This study adhered to the protocol approved by the Institutional Animal Care and Use Committee at the National Institutes of Health.

Note that full information on the approval of the study protocol must also be provided in the manuscript.

Human research participants

Policy information about studies involving human research participants

Population characteristics	The age of participants was 35.5 +/- 12.8 (mean +/- SD) with 6 females and 5 males (see Table S1 for additional

Population characteristics	characteristics).
Recruitment	Subjects were recruited from the National Eye Institute Eye Clinic at the National Institutes of Health, Bethesda, Maryland. Informed consent was obtained from all participants prior to enrollment. To be eligible for inclusion, subjects had to be at least 12 years of age (at least 18 years of age for those subjects undergoing ICG). Subjects were excluded from participation if they had a known allergy to ICG, iodine, or shellfish, were pregnant or nursing, or were participating in any other investigational drug study.
Ethics oversight	This study was approved by the institutional review board of the National Institutes of Health and was conducted in accordance with the Declaration of Helsinki.

Note that full information on the approval of the study protocol must also be provided in the manuscript.
