# Supplementary material for: Widespread subclinical cellular changes revealed across a neural-epithelial-vascular complex in choroideremia using adaptive optics
Source: Commun Biol. 2022 Sep 13;5:893. doi: 10.1038/s42003-022-03842-7 (PMC9470576; doi:10.1038/s42003-022-03842-7)
Supplement: Supplementary file 1 — Peer Review File [file 42003_2022_3842_MOESM1_ESM.pdf]

nature portfolio

Peer Review File

Widespread subclinical cellular changes revealed across a neural-epithelial-vascular complex in choroideremia using adaptive optics

Open Access This file is licensed under a Creative Commons Attribution 4.0 International License, which permits use, sharing, adaptation, distribution and reproduction in any medium or format, as long as you give appropriate credit to the original author(s) and the source, provide a link to the Creative Commons license, and indicate if changes were made. In the cases where the authors are anonymous, such as is the case for the reports of anonymous peer reviewers, author attribution should be to 'Anonymous Referee' followed by a clear attribution to the source work. The images or other third party material in this file are included in the article's Creative Commons license, unless indicated otherwise in a credit line to the material. If material is not included in the article's Creative Commons license and your intended use is not permitted by statutory regulation or exceeds the permitted use, you will need to obtain permission directly from the copyright holder. To view a copy of this license, visit http://creativecommons.org/licenses/by/4.0/.

Reviewers' comments:

Reviewer #2 (Remarks to the Author):

Thank you for opportunity of reading and reviewing this manuscript. Having considered patient communication to be of utmost importance to this subject, the paper reaffirms my conviction that we must teach the skills of how to communicate effectively. The subject is important to both patients and clinicians as both need information. I would welcome their thoughts and recommendations about how to better inform patients and their clinicians about the risks and benefits of ocular gene therapy. Viral mediated gene therapy is not without risk especially as it related to inflammation. Some patients do not even realize that gene therapy can be gene or mutation specific.

General comments:
1. the authors use "widespread" as a category of IRDs. "Generalized" would be more acceptable in my opinion.
2. Line 216: Parents and caregivers had more information needs and low negative expectations. Would the authors care to speculate as to why?
3. Line 251. The phrasing is awkward. The sentence reads as if knowledge gaps are critically important... when we should be closing the gaps.
Table 2: Would the authors consider re-arranging the columns to put a more positive spin on the date. The first column would then be Agree, the Neither, then Disagree. Then rank the responses under Agree with a. = Internet, b. = my ophthalmologist, etc. Your ophthalmologists appear to be doing a great job!

Reviewer #3 (Remarks to the Author):

General Comments:
Aguilera at al use an innovative method employing late-stage ICG imaging in an AOSLO to reveal the RPE mosaic in CHM-affected and CHM-carriers with comparisons to similar measurements in healthy normal eyes. The main result is the discovery of extensive RPE-cell enlargement and RPE-cell polymegathism in affected eyes and in the female carriers.
They also find that, in some cases, the cones uptake the ICG dye and fluoresce also indicating that RPE photoreceptor barrier might be compromised. The results using this innovative ICG imaging approach are corroborated by AO-OCT in the same eyes as well as with animal studies. The authors make an important point that some of the ICG imaging results can be done using commercial SLOs that do not use adaptive optics. Overall, the study is thorough and very well executed. The paper is very well written.

One of the most surprising findings is the extent to which female carriers of the CHM mutation are affected. The RPE cells in carriers are surprisingly large and irregular yet, despite this, the cone functon(measure using a microperimeter) appears normal. I think there is an opportunity for the authors to discuss this more, and perhaps search the literature a bit more on what functional deficits these female carriers exhibit. I find it hard to believe that there can be no consequences of such significant RPE cell differences.

Overall, the results make important new discoveries for this disease that will have an impact, especially considering that several gene therapy trials are underway and more are planned in the future.

Specific comments:

There are a series of minor comments that, if addressed, will improve this already excellent paper.

Lines 111-112: The RPE cells in the AO-OCT images in Fig 2A are not clear at all. Maybe it is the figure reproduction, but I suspect it is due a limit of the modality itself (considering that the other images look great). This is unfortunate, considering that this is one of the more important corroborations that could be made in this paper. Are there any more compelling example? Or maybe it is because the image to too magnified? This is not a fatal flaw, but the authors might be a bit more forthcoming about the challenges of AO-OCT for imaging RPE cell in patients with advanced disease.

Lines 193-201: The authors should investigate and report if there are any quantifiable differences in the RPE mosaic (cell size, polymegathism, ICG uptake) underlying the fluorescent photoreceptor cells compared to other areas.

Lines 367-368: How many images were discarded?

Response to Reviewer Comments

The authors would like to thank the editors and reviewers for their insightful comments and suggestions.

Reviewer #2 comments (truncated after consultation with the Editors):

Thank you for opportunity of reading and reviewing this manuscript. Having considered patient communication to be of utmost importance to this subject, the paper reaffirms my conviction that we must teach the skills of how to communicate effectively. The subject is important to both patients and clinicians as both need information. I would welcome their thoughts and recommendations about how to better inform patients and their clinicians about the risks and benefits of ocular gene therapy. Viral mediated gene therapy is not without risk especially as it related to inflammation. Some patients do not even realize that gene therapy can be gene or mutation specific.

Editor suggestion: Include 2-3 sentences in the discussion that address Referee 2's broad concern about patients' information needs for the outcome of clinical evaluations for choroideremia

Author response: We agree with the need to address patients' information needs for the outcome of clinical evaluations for choroideremia. We have added a citation to important recent research report which discusses this in more detail, and have also added a few sentences to the second to last paragraph of the discussion to emphasize this point:

"We expect that visualization of the RPE at the cellular level will have a positive impact on current and future gene therapy trials, as a means to better inform patients of their status and eligibility for treatment, to monitor possible risks as treatment trials progress, and also as a potential outcome of clinical evaluations for choroideremia[54]. Ultimately, including female carriers who exhibit signs of RPE disease alongside affected males in such trials[24] could help to accelerate progress towards realizing a cure for the disease."

Reviewer #3 comments

Aguilera at al use an innovative method employing late-stage ICG imaging in an AOSLO to reveal the RPE mosaic in CHM-affected and CHM-carriers with comparisons to similar measurements in healthy normal eyes. The main result is the discovery of extensive RPE-cell enlargement and RPE-cell polymegathism in affected eyes and in the female carriers.

They also find that, in some cases, the cones uptake the ICG dye and fluoresce also indicating that RPE photoreceptor barrier might be compromised. The results using this innovative ICG imaging approach are corroborated by AO-OCT in the same eyes as well as with animal studies. The authors make an important point that some of the ICG imaging results can be done using commercial SLOs that do not use adaptive optics. Overall, the study is thorough and very well executed. The paper is very well written.

One of the most surprising findings is the extent to which female carriers of the CHM mutation are affected. The RPE cells in carriers are surprisingly large and irregular yet, despite this, the cone functon(measure using a microperimeter) appears normal. I think there is an opportunity for the authors to discuss this more, and perhaps search the literature a bit more on what functional deficits these female carriers exhibit. I find it hard to believe that there can be no consequences of such significant RPE cell differences.

Overall, the results make important new discoveries for this disease that will have an impact, especially considering that several gene therapy trials are underway and more are planned in the future.

Author response: Thank you for the positive feedback. We agree that it is surprising to find the degree of RPE disruption observed in the female carriers. However, for female carriers of CHM, the structural deficits are not always associated with functional ones. As suggested, we have modified the third paragraph of the introduction by expanding on the symptomology exhibited by female carriers in relation to structural signs of disease observed through ophthalmoscopy:

"Female carriers of choroideremia are typically regarded to be mostly unaffected. Although it is recognized that there is a wide spectrum of disease severity affecting female carriers, severe symptoms such as night blindness and visual impairment similar to those observed in affected males are thought to affect only a minority of the female carriers[24]. In a previous study, abnormal full-field electroretinography responses were detected in only 15% of female carriers, despite 96% of these carriers presenting with ophthalmoscopic signs such as patchy atrophy and coarse granularity in the mid and far periphery[25]. It has been hypothesized that the degree to which female carriers are affected depends on lyonization (random X-inactivation)[12,16,26,27] within the RPE mosaic, which would result in patches of both mutant and normal RPE cells in which the mutant RPE cells could degenerate over the course of five or more decades of life. In particular, lyonization could explain the patchy pigmentary changes such as melanin clumping due to pigment migration or areas of RPE atrophy reported in female carriers[7,12,28-30]."

In the third paragraph of the discussion, we have added text describing a histological study that reinforces our observations of enlarged RPE in female carriers:

"Our data is consistent with histological findings of polymegathism of the RPE observed in a female carrier[51]."

We have also added a sentence to the end of this same paragraph to indicate the uniformity with which we observed these changes across the cohort of female carriers:

"This late phase ICG pattern of patchy cyanescence appears to be a characteristic pattern, observed in 100% of the female carriers imaged."

There are a series of minor comments that, if addressed, will improve this already excellent paper.

Lines 111-112: The RPE cells in the AO-OCT images in Fig 2A are not clear at all. Maybe it is the figure reproduction, but I suspect it is due a limit of the modality itself (considering that the other images look great). This is unfortunate, considering that this is one of the more important corroborations that could be made in this paper. Are there any more compelling example? Or maybe it is because the image to too magnified? This is not a fatal flaw, but the authors might be a bit more forthcoming about the challenges of AO-OCT for imaging RPE cell in patients with advanced disease.

Author response: We agree with the reviewer's assessment of the limitations of AO-OCT, particularly with respect to eye motion which affects the AO-OCT channel more than the AO-SLO channel because of its slow volume rate. In general, the affected males had noticeably more eye motion that contributed to the challenge of imaging subjects with this disease. However, it is not clear that the appearance of the RPE cells in Fig. 2A is due solely to the modality and may also be from the disease itself. Although the images of RPE cells in carriers show dark spots that are spaced apart to a greater extent compared to those in healthy eyes, in general, due to the dramatic enlargement in RPE cell size, in the affected males the AO-OCT RPE images have relatively few RPE cell centers present within each image. The same field-of-view (FOV) was used to obtain AO-OCT RPE images from both healthy and diseased eyes: all panels of Fig. 2 are showing the full AO-OCT image, so no artificial magnification is applied to display the AO-OCT images. To address these points, we have added the following sentences to the revised manuscript:

"Although AO-OCT imaging in healthy eyes has been successfully demonstrated, to our knowledge, there are limited examples of AO-OCT imaging performed in patients with advanced disease, which can be challenging due to the longer acquisition time needed to obtain averaged volumes for RPE cell imaging (125-150 volumes per location; see Methods: Adaptive Optics Imaging)."

and

"In general, the sources of image contrast are not yet well understood when the cellular structure itself is as dramatically altered as it is in the case of these enlarged RPE cells, which introduces challenges for image interpretation. Nonetheless, these multimodal AO images provide an initial glimpse into the characteristics of AO-OCT RPE imaging in diseased eyes and help to confirm that the RPE monolayer is intact even in areas of low cell contrast (AO-OCT shows the presence of RPE cells in hypofluorescent areas of the AO-ICG image, and conversely, AO-ICG shows the presence of RPE cells in low-contrast areas of the AO-OCT image). "

We recognize that there are important steps that need to be taken to translate a technique that has been demonstrated in healthy eyes to patients with advanced disease, and we are hopeful that these initial examples provide a first step towards establishing how best to interpret AO-OCT RPE images in disease eyes.

Lines 193-201: The authors should investigate and report if there are any quantifiable differences in the RPE mosaic (cell size, polymegathism, ICG uptake) underlying the fluorescent photoreceptor cells compared to other areas.

Author response: This is a very insightful comment, because evaluating whether there are disruptions to the RPE underlying areas of fluorescent photoreceptors could help to explain this phenomenon. Unfortunately, it is very difficult to assess the RPE underlying these areas because the fluorescence signal of the photoreceptors is superimposed on top of the fluorescence signal of the RPE. Since we are unable to separate the two signals, we cannot make a measurement of ICG uptake into the RPE alone; we can only measure the combined photoreceptor + RPE fluorescence. Close examination of Supp Fig 1 shows that there is variability in the degree to which the photoreceptors have been labeled by ICG. Due to this variability, it is difficult to quantify ICG uptake by the RPE. Likewise, due to the RPE mosaic being obscured by the fluorescent photoreceptors, it is also quite difficult to determine the locations of RPE cells underlying fluorescent photoreceptors.

That said, we do believe it is important to explore the status of RPE underlying fluorescent photoreceptors. To address this, we have added two new observations to the revised manuscript.

First, we observed that many of the regions with fluorescent photoreceptors appeared to be in the proximity of small vitelliform-like lesions (**Fig. S3**). We re-examined the OCT b-scans and fundus autofluorescence images in regions identified to have fluorescent photoreceptors, in order to determine if each of these regions were associated with vitelliform-like lesions. We found that half (50%) of the regions with fluorescently-labeled photoreceptors were associated with a neighboring vitelliform-like lesion. These lesions appeared to be of adult onset when comparisons were made with previous multimodal imaging from the same patients. We therefore hypothesize that these vitelliform-like lesions are secondary to the underlying retinal degeneration (choroideremia) and are a further indication of RPE dysfunction. We also note that

the vitelliform material blocks the ICG fluorescence signal (hypofluorescent appearance), which further complicates any measurements of ICG uptake into the RPE near these regions. We have added following text about these findings to the second to last paragraph of the "Disruption of outer retinal barrier function of the RPE" section:

"Further examination of OCT scans and fundus autofluorescence in patches of fluorescently labeled photoreceptors revealed that half (50%) of the patches were associated with a neighboring vitelliform-like lesion (typically hypocyanescent due to blocking of the ICG fluorescence). These lesions appeared to be of adult onset when comparisons were made with previous multimodal imaging from the same patients. We therefore hypothesize that these vitelliform-like lesions are secondary to the underlying retinal degeneration (choroideremia) and are a further indication of RPE dysfunction."

Second, we have added a new supplemental figure (**Fig. S2**). As described above, it is difficult to clearly discern the locations of RPE cells underlying fluorescent-labeled photoreceptors using AO-ICG. However, in one patient, we were able to obtain high quality darkfield images. Although this is only one example, qualitatively, it does appear that the RPE cells underlying a patch of fluorescently-labeled photoreceptors are enlarged when compared to the neighboring RPE mosaic (unfortunately, the darkfield images in the remaining patients did not reveal a clear RPE mosaic). The difference in RPE size cannot be attributed to eccentricity-dependent differences. Notably, this example represents a patch that is not neighboring a vitelliform-like lesion, further supporting our claim that the fluorescent labeling of photoreceptors arises from an RPE defect. Given that we were not able to find additional examples, we opted not to quantify this darkfield image so as not to overstate the claim. We have added the following text about this finding to the second to last paragraph of the "Disruption of outer retinal barrier function of the RPE" section:

*"Unfortunately, it was difficult to assess RPE cells underlying patches of fluorescently labeled photoreceptors as the fluorescent signal from the photoreceptors obscured the RPE mosaic (**Fig. S1**). However, in one patient, simultaneously-acquired darkfield images of the RPE mosaic[46] were visible in which there appeared to be RPE enlargement underlying a patch of fluorescently-labeled photoreceptors (**Fig. S2**). This degree of RPE enlargement cannot be explained by eccentricity-dependent differences in RPE morphology[40], which would support the theory that these patches arise due to defects in the RPE."*

Fig. S2, which has been added to the revised manuscript, is reproduced below:

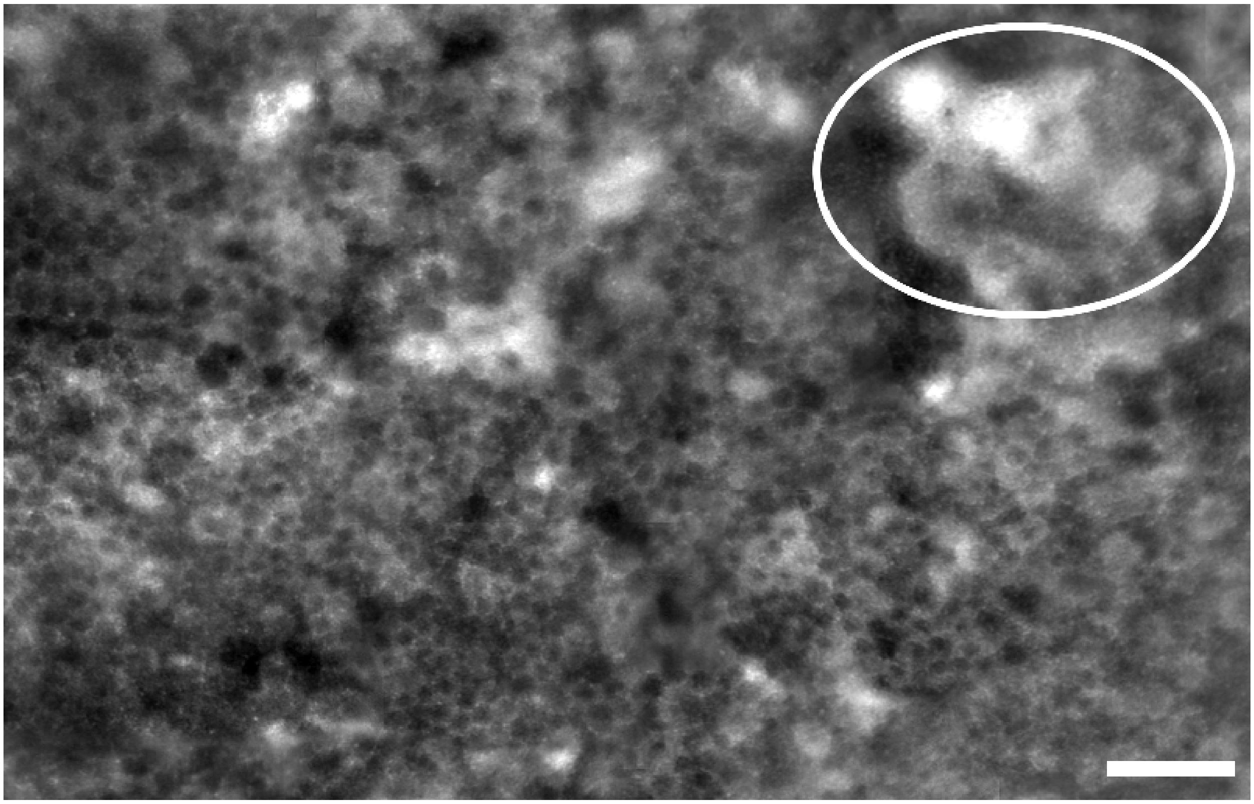

Fig. S2. Darkfield RPE imaging in Subject C2L. The circled area represents a patch of fluorescently labeled photoreceptors. The pattern of RPE cells appears to be enlarged within the circled area compared to the surrounding regions of the image. There is no vitelliform-like lesion neighboring this patch. Scale bar, 100 µm.

Lines 367-368: How many images were discarded?

Author response: We want to acknowledge that not all of the ROIs selected were used. Out of a total of 125 ROIs, 7 were discarded. This was due to the images having large areas of hypofluorescence, making it difficult for the graders to agree on the locations of RPE cells within the areas of hypofluorescence. We have added the following sentence to the methods in the revised manuscript:

"Out of a total of 125 ROIs selected, 7 containing large areas of hypofluorescence that made grading difficult were discarded (6% of the ROIs)."

Other Minor Changes

In addition to the changes described above, we have made some corrections to the text, added references, and updated some descriptions to be consistent with the Nature Reporting Summary forms.

- In the 1st paragraph of the "Disruption of outer retinal barrier function of the RPE", we added a citation to a publication describing the custom AO microscope used for imaging [Giannini 2022].

- In the 5th paragraph of the discussion, we added a citation to a publication that is similar to the patient shown in Figure 5A:

  "Our findings also help to explain the smooth and mottled pattern of autofluorescence that has been previously reported in some affected males [Stevanovic 2020]."

- The caption for Figure 3B was updated to indicate the box plot elements:

  "**(B)** Box plots of photoreceptor spacing, RPE spacing, and choriocapillaris flow void diameters show that the RPE is the most severely affected layer of these three layers (center line: median; box limits: upper/lower quartiles; whiskers: 1.5x interquartile range; points beyond the whiskers: outliers). …"

- Edited acknowledgements: "Furu Zhang for assistance with adaptive optics image processing"

- Re-arranged supplemental numbering to account for new supplemental Fig. S2. Darkfield RPE imaging in Subject C2L

- Figure S3B was updated to add a second vitelliform-like lesion that was identified upon re-examination of the imaged areas alongside OCT and FAF (dashed circle superior to the fovea).

- We corrected a misspelling in Table S2. X-inactivation, changing "subjet ID" to "Subject ID".
